# DUSP4 modulates RIG-I- and STING-mediated IRF3-type I IFN response

**DOI:** 10.1038/s41418-024-01269-7

**Published:** 2024-02-21

**Authors:** Huipeng Jiao, Sharmy J. James, Chin Wen Png, Chaoyu Cui, Heng Li, Liang Li, Wan Ni Chia, Nyo Min, Weiyun Li, Carla Claser, Laurent Rénia, Hongyan Wang, Mark I-Cheng Chen, Justin Jang Hann Chu, Kevin Shyong Wei Tan, Yinyue Deng, Yongliang Zhang

**Affiliations:** 1 Zhejiang Provincial Key Laboratory of Cancer Molecular Cell Biology, Life Sciences Institute, Zhejiang University, Hangzhou, Zhejiang, 310058 China; 2 Department of Microbiology and Immunology, TRP Immunology, Yong Loo Lin School of Medicine, National University of Singapore, Singapore, 117597 Singapore; 3 Immunology Programme, Life Sciences Institute, National University of Singapore, Singapore, 117597 Singapore; 4 School of Pharmaceutical Sciences (Shenzhen), Shenzhen Campus of Sun Yat-sen University, Sun Yat-sen University, Shenzhen, 518100 China; 5 Department of Pharmacology, School of Medicine, Southern University of Science and Technology, Shenzhen, 518055 China; 6 State Key Laboratory of Cell Biology, Shanghai Institute of Biochemistry and Cell Biology, Chinese Academy of Sciences, University of Chinese Academy of Sciences, Innovation Center for Cell Signaling Network, Shanghai, 200031 China; 7 Singapore Immunology Network, Agency for Science, Technology and Research (A*STAR), Singapore, 138668 Singapore; 8 Saw Swee Hock School of Public Health, National University of Singapore, Singapore, 117597 Singapore

**Keywords:** Signal transduction, Inflammation

## Abstract

Detection of cytosolic nucleic acids by pattern recognition receptors, including STING and RIG-I, leads to the activation of multiple signalling pathways that culminate in the production of type I interferons (IFNs) which are vital for host survival during virus infection. In addition to protective immune modulatory functions, type I IFNs are also associated with autoimmune diseases. Hence, it is important to elucidate the mechanisms that govern their expression. In this study, we identified a critical regulatory function of the DUSP4 phosphatase in innate immune signalling. We found that DUSP4 regulates the activation of TBK1 and ERK1/2 in a signalling complex containing DUSP4, TBK1, ERK1/2 and IRF3 to regulate the production of type I IFNs. Mice deficient in DUSP4 were more resistant to infections by both RNA and DNA viruses but more susceptible to malaria parasites. Therefore, our study establishes DUSP4 as a regulator of nucleic acid sensor signalling and sheds light on an important facet of the type I IFN regulatory system.

## Introduction

The presence of nucleic acids in the cytosol is a danger signal that triggers robust innate immune response in mammalian cells [1]. Cytoplasmic nucleic acids, including RNA and DNA, are detected by various germline-line encoded pattern recognition receptors (PRRs) that activate signalling cascades, mainly the interferon regulatory factors (IRFs), the mitogen-activated protein kinases (MAPKs) and the nuclear factor kappa B (NFκB) pathways, leading to expression of antimicrobial effector molecules and activation of sentinel immune cells. For instance, the cytosolic retinoic acid inducible gene I (RIG-I)-like receptors, including RIG-I, MDA5 and LGP2, recognise viral RNA to activate antiviral immunity [2, 3]. Particularly, RIG-I detects double-stranded RNA derived from RNA viruses, including influenza virus, Japanese encephalitis virus, Sendai virus (SeV) and vesicular stomatitis virus (VSV), to trigger robust antiviral immunity [3–5]. The cGAS-STING pathway, on the other hand, is central for the detection of cytosolic DNA and the ensuing immune responses to infection with DNA viruses such as herpes simplex virus-1 (HSV-1) and Kaposi’s sarcoma-associated herpes virus (KSHV) [6–8]. The detection of cytosolic nucleic acids by aforementioned PRRs activates signalling pathways that culminate in the expression of type I interferons (IFNs) including IFNα and IFNβ.

Type I IFNs are imperative for antiviral defence. Mice lacking a functional type I IFN system are unable to cope with viral infections [9, 10]. Similarly, inherited impairment of the type I IFN system in humans results in lethal viral diseases [11–13]. On the other hand, type I IFNs may inhibit host defence to infection under certain circumstances. For instance, influenza-induced type I IFNs sensitise hosts to secondary bacterial infection, which could lead to bacterial superinfections and lethality [14]. In addition, type I IFNs induced by nonviral pathogens, including *Listeria*, *Mycobacteria* and *Plasmodium*, increase host susceptibility to infection [15–17]. Furthermore, inappropriate upregulation of type I IFN activity is associated with pathogenesis of autoimmune diseases such as Aicardi–Goutières syndrome and systemic lupus erythematosus [18]. Intriguingly, type I IFN expression stimulated by tumour-derived DNA via STING promotes anti-tumour immune responses [19, 20]. The wide range of physiological functions of type I IFNs underscores the complexity of the type I IFN system. A better understanding of the processes governing the expression of type I IFNs will help in developing new approaches for prevention and treatment of diseases involving type I IFNs.

Various PRR signalling pathways converge at key molecules including the Tank-binding kinase (TBK) 1 and the IRF family of transcription factors to activate the transcription of genes encoding IFNα/β [21]. For example, the RIG-I-MAVS and cGAS-STING pathways are interconnected and converged at TBK1 that directly phosphorylates IRF3, the primary transcriptional factor regulating the induction of type I IFNs [22]. TBK1 and IKKε are the two established IRF3 kinases. The expression of TBK1 is ubiquitous [23], whereas IKKε is inducible in lymphoid and other cell types [24]. These two kinases phosphorylate serine residues in the C-terminus of IRF3 for its activation and transcription activity [25, 26]. In addition, several protein kinases, including DNA-PK, JNK and ERK, have been implicated in the phosphorylation of IRF3, thereby contributing to its activation [27–30]. However, the roles of these kinases in the activation and function of the IRF3-type I IFN system remain inconclusive.

The activation and deactivation of signalling molecules must be finely regulated, often by phosphorylation and dephosphorylation events [31]. The dual-specificity MAPK phosphatases (MKPs) are a subgroup of dual-specificity protein phosphatases (DUSPs), capable of dephosphorylating tyrosine, threonine and serine residues in substrates to regulate the activation of important signalling molecules, including MAPKs, IRF3, and possibly STAT3/5 [32, 33]. However, the physiological functions of DUSPs are yet to be appreciated.

In this report, we demonstrate that DUSP4 regulates the signalling events activated by cytoplasmic nucleic acids from microbial organisms. This protein participates in the assembly of a signalling complex that contains ERK1/2, TBK1, and IRF3 in response to stimulation by various cytoplasmic PRRs, including RIG-I and STING, thereby regulating the activation of ERK1/2 and TBK1. It regulates the recruitment of ERK1/2 and TBK1 to the signalling complex to control the activation of IRF3. Additionally, we provided evidence supporting the possibility of ERK1/2 serving as IRF3 kinases, thereby directly regulating its activation. Therefore, DUSP4 may target both TBK1 and ERK1/2 to regulate the IRF3-type I IFN system.

## Results

### DUSP4 expression in macrophages suppresses RIG-I- and TLR3-mediated activation of ERK and TBK1-IRF3 signaling

**Fig. 1:**
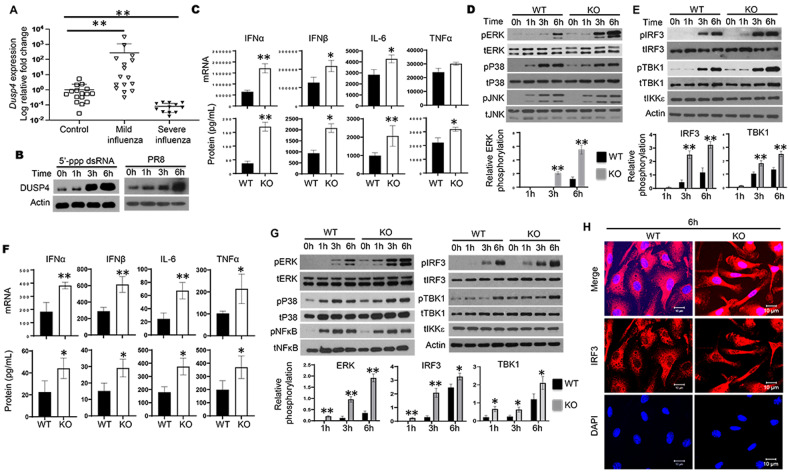
Increased activation of ERK and TBK1-IRF3, and increased expression of cytokines in DUSP4 KO macrophages in response to RIG-I activation and influenza infection. **A** Expression of *DUSP4* in PBMCs from healthy individuals and patients with mild influenza (*n* = 16), and patients with severe influenza (*n* = 12) was analysed by quantitative real-time PCR (qPCR). Severe disease was defined as evidence of pulmonary involvement on chest radiography or need for supplemental oxygen during admission or both, while mild disease was absence of both criteria for severity. Mann-Whitney non-parametric test was use to perform statistical analysis. **P < 0.01. **B** Immunoblot analysis of DUSP4 protein expression in bone marrow-derived macrophages (BMDMs) from wildtype (WT) mice upon 5′-ppp dsRNA stimulation (0.5 μg/mL), or infected with PR8 influenza virus at a multiplicity of infection of 1. **C** Expression of IFNα, IFNβ, IL-6 and TNFα mRNA in WT and knockout (KO) BMDMs at 3 h after 5′-ppp dsRNA stimulation (0.5 μg/mL) was determined by qPCR. Cytokines in culture supernatants of WT and KO BMDMs at 6 h (for IFNα and IFNβ) or 24 h (for IL-6 and TNFα) after 5′-ppp dsRNA stimulation (0.5 μg/mL) were determined by ELISA. Activation of ERK, JNK and p38 (**D**), and TBK1, IKKε and IRF3 (**E**) in WT and KO BMDMs at various time-points after 5’-ppp dsRNA stimulation (0.5 μg/mL) was assessed by immunoblot analysis. The phosphorylation levels of TBK1 and IRF3 (*n* = 3) were quantified using ImageJ. **F** Cytokine expression in WT and KO BMDMs in response to influenza H1N1 PR8 virus infection (MOI of 1) at mRNA level at 6 h or at protein level at 6 h (for IFNα and IFNβ) or 24 h (for IL-6 and TNFα) post infection (PI) was determined by qPCR or ELISA respectively. **G** Increased activation of ERK, TBK1 and IRF3 in DUSP4 KO BMDMs at various time-points after PR8 infection at an MOI of 1. The phosphorylation levels of ERK, TBK1 and IRF3 (*n* = 3) were quantified using ImageJ. **H** Confocal microscopy of WT and KO BMDMs showing increased IRF3 nuclear accumulation compared to WT cells. Nuclei were stained with DAPI. Unpaired *T*-test was used for statistical analysis. *P < 0.05; **P < 0.01. Data are representative of at least three independent experiments with similar results.

DUSP4, also known as MKP2, has been shown to regulate immune response to *Leishmania* parasite infection [34]. However, its role in immune response to viral infection is unknown. To study the function of DUSP4 in immune response to viral infection, we analysed the expression of *DUSP4* in PBMCs from patients with mild or severe H1N1 influenza disease. Compared to healthy controls, *DUSP4* expression was increased in PBMCs from patients with mild influenza, but decreased in PBMCs from patients with severe influenza (Fig. 1A). These results suggest that the level of *DUSP4* expression in PBMCs from influenza patients may be associated with disease severity.

To investigate the possible involvement of this gene in antiviral response, we transfected GFP-tagged murine *Dusp4* cDNA construct into RAW264.7 cells, and found that DUSP4 is localised in both the cytoplasm and nuclear, but is primarily localised in cytoplasm of non-infected cells (Fig. S1). Upon VSV infection, significantly increased accumulation of DUSP4 in the nucleus was detected.

To examine the involvement of DUSP4 in PRR signalling, C57BL/6 wildtype (WT) bone marrow-derived macrophages (BMDMs) were stimulated with 5′-ppp dsRNA, a synthetic ligand for RIG-I. It was found that DUSP4 protein was constitutively expressed in BMDMs at a low level, and its expression was increased at 3 and 6 h post stimulation (Fig. 1B). For various cytokines including IFNα, IFNβ, IL-6 and TNFα, their expression was undetectable in both WT and KO BMDMs without stimulation. Expression of these cytokines was observed in WT cells in response to the stimulation and increased expression of IFNα, IFNβ, IL-6 and TNFα was detected in DUSP4 knockout (KO) cells compared to WT cells (Fig. 1C). Immunoblot analysis for the activation of MAPKs, namely extracellular signal-regulated kinase 1/2 (ERK1/2), c-Jun N-terminal kinase (JNK) and p38, revealed increased activation of ERK1/2 but not p38 or JNK in KO cells at 3 and 6 h post stimulation (Fig. 1D). On the other hand, NFκB activation between WT and KO cells was comparable (Fig. S2A). These results indicate that DUSP4 specifically targets ERK1/2 in macrophages in response to RIG-I activation. Interestingly, the activation of TBK1 and IRF3 was also increased in KO cells compared to that in WT cells (Fig. 1E). Similarly, in response to TLR3 stimulation, DUSP4 KO BMDMs expressed higher levels of IFNα, IFNβ and IL-6 than WT cells (Fig. S2B), correlating with increased activation of ERK1/2, TBK1 and IRF3 (Fig. S2C). Together, these results suggest that DUSP4 plays an important role in the regulation of cytokine expression upon the detection of viral RNA.

In addition, increased expression of DUSP4 protein was detected at 3 and 6 h in WT BMDMs infected with influenza H1N1 A/Putero Rico/8/1934 (PR8) viruses (Fig. 1B). Further analysis showed that DUSP4 KO BMDMs had increased expression of IFNα, IFNβ, IL-6 and TNFα after PR8 infection (Fig. 1F), which was associated with increased activation of ERK1/2, TBK1 and IRF3 compared to WT cells (Fig. 1G). Increased expression of interferon-stimulated genes (ISGs), including RANTES, ISG15 and 2’-5’-oligoadenylate synthetase (2’-5’-OAS), was also detected in KO cells compared to WT cells (Fig. S3A). Furthermore, enhanced nuclear accumulation of IRF3, an essential step for IRF3 transcriptional activity, was observed in KO BMDMs upon PR8 infection compared to that in WT cells (Fig. 1H and Fig. S3B), confirming the increased activation of IRF3 in KO BMDMs in response to influenza infection. In addition, increased expression of type I IFNs and proinflammatory cytokines including IL-6 and TNFα was observed in DUSP4 KO bone marrow-derived dendritic cells (BMDCs) compared to WT cells upon PR8 infection (Fig. S3C). We also detected increased expression of IFNα, IFNβ, IL-6 and TNFα in DUSP4 KO BMDMs in response to SeV infection (Fig. S4A) or VSV infection (Fig. S4B) compared to WT cells. Together, these results demonstrate that DUSP4 inhibits the activation of ERK1/2 and TBK1-IRF3 signalling in macrophages in response to RNA virus infection to inhibit the expression of type I IFNs and proinflammatory cytokines.

### Deficiency of DUSP4 in mice resulted in resistance to influenza infection

**Fig. 2:**
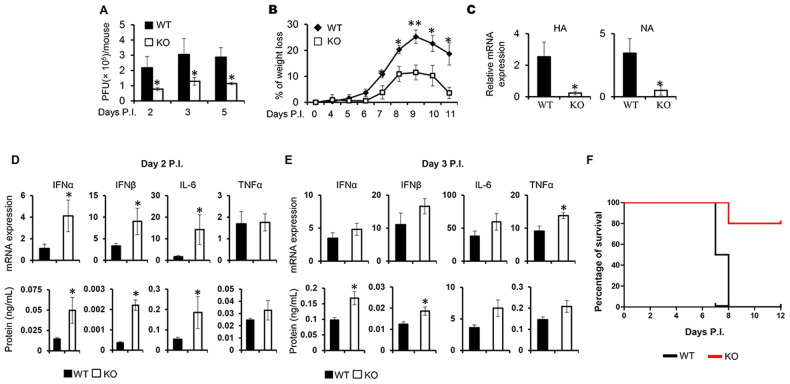
DUSP4 KO mice were more resistance to influenza infection compared to WT mice. **A** WT and DUSP4 KO mice were infected with 50 plaque-forming units (PFU) of PR8 influenza virus intranasally. Viral titers in the lung of WT and DUSP4 KO mice (*n* = 3 for each time point) at day 2, 3 and 5 PI were analysed by plaque assay. **B**, **C** WT and KO mice (*n* = 5) were infected with 50 PFU of PR8 virus. Changes in body weight were monitored daily (**B**). Expression of influenza hemagglutinin (HA) and neuraminidase (NA) in WT and DUSP4 KO mice (*n* = 5) on day 11 PI was analyzed by qPCR (C). **D**, **E** On day 2 or 3 PI, lungs were harvested from WT and KO mice (*n* = 5). Cytokine mRNA expression and protein concentrations in lung homogenates were measured by qPCR and ELISA respectively. **F** WT and DUSP4 KO female mice (*n* = 5) were infected with a lethal dose (150 PFU) of H1N1 PR8 influenza viruses. The survival of the mice was monitored daily post infection (PI) (Log-rank test, *P* = 0.034). Unpaired *T*-test was used for statistical analysis. *P < 0.05; **P < 0.01. Data are representative of at least three independent experiments with similar results.

To validate the function of DUSP4 in response to virus infection in vivo, both WT and KO mice were infected with a sublethal dose of PR8 viruses. Results showed that the KO mice had reduced viral loads in the lung on day 2, 3, and 5 post infection (PI) (Fig. 2A), and developed less severe disease compared to WT mice over the course of infection (Fig. 2B). In addition, the expression of hemagglutinin and neuraminidase genes in the lungs of KO mice on day 11 PI was significantly lower than that in WT mice (Fig. 2C). These results demonstrate that DUSP4 KO mice are more resistant to H1N1 influenza infection than WT mice. This increased anti-influenza capability of the KO mice was associated with an elevated expression of IFNα and IFNβ, as well as IL-6 and TNFα in the lung, compared to WT mice (Fig. 2D, E). Furthermore, increased survival of the KO mice compared to WT mice in response to lethal influenza infection was observed (Fig. 2F). Majority of the WT mice reached the ethical endpoint requiring euthanasia before day 8 post-infection, whereas about 80% of the KO mice survived. Together, these data indicate that DUSP4 negatively regulates the innate immune response to influenza infection.

### DUSP4 suppresses STING-mediated activation of ERK and TBK1-IRF3 signalling

**Fig. 3:**
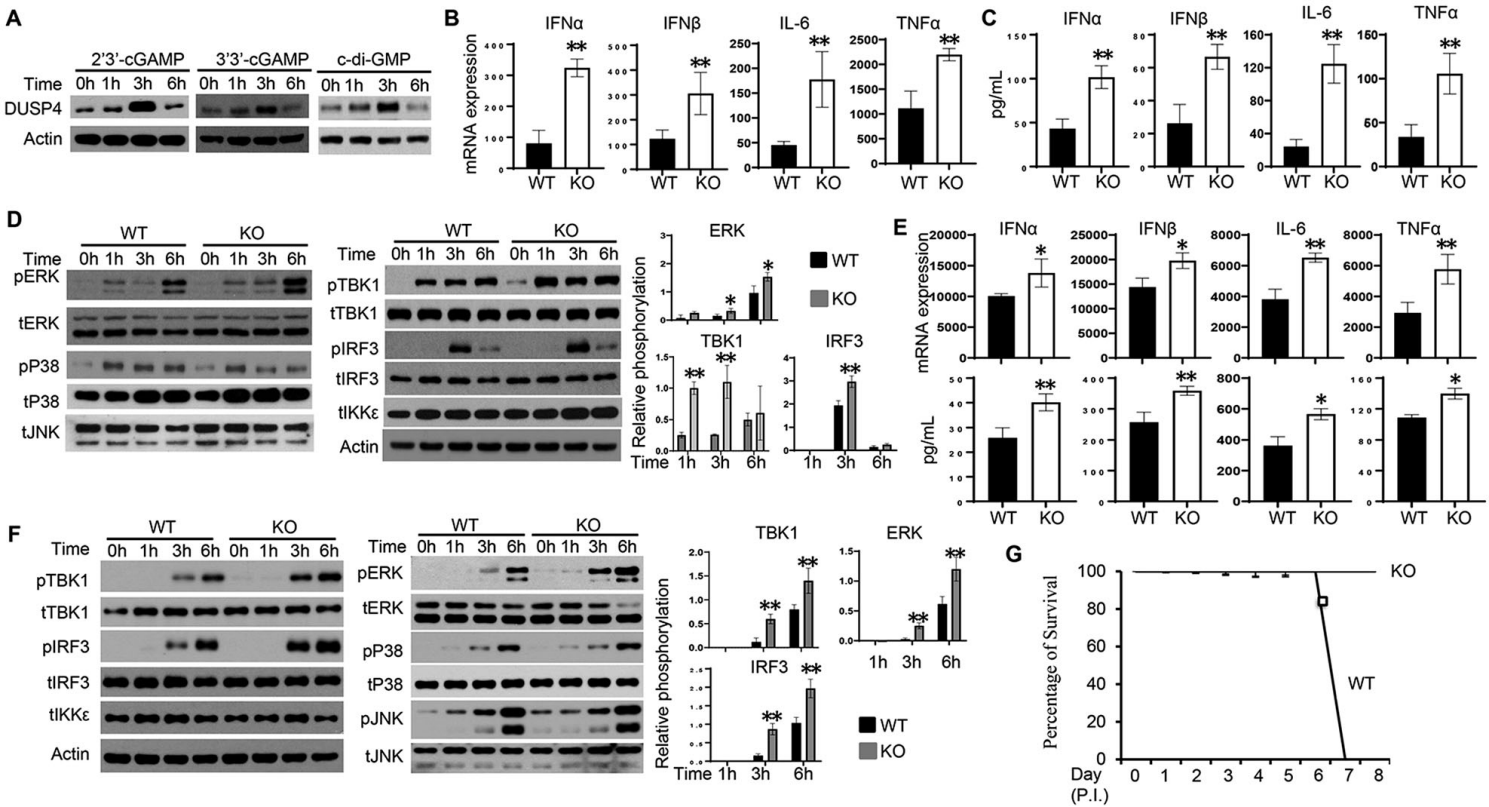
Increased ERK and TBK1-IRF3 activation and cytokine expression in DUSP4 KO macrophages in response to STING stimulation and HSV-1 infection. **A** WT BMDMs were transfected with 0.5 μg/mL of 2′3′-cGAMP, 3′3′-cGAMP or c-di-GMP. Cells were harvested at the indicated time-points to assess DUSP4 expression by immunoblot analysis. **B**, **C** WT and KO BMDMs were stimulated with c-di-GAP (0.5 μg/mL) for 3 h to examine the expression of IFNα, IFNβ, IL-6 and TNFα by qPCR (**B**), 6 h (for IFNα and IFNβ) or 24 h (for IL-6 and TNFα) to determine protein concentrations of cytokines in culture supernatants by ELISA (**C**). **D** WT and KO BMDMs were transfected with 0.5 μg/mL of c-di-GMP. Cells were harvested at the indicated time points to examine the activation of ERK, JNK and p38 (activation of JNK was undetectable), and TBK1, IKKε, IRF3 and NFκB by immunoblot analysis. The phosphorylation levels of ERK, TBK1 and IRF3 (*n* = 3) were quantified using ImageJ. **E**, **F** WT and DUSP4 KO BMDMs were infected with HSV-1 at an MOI of 0.01 for 6 h to examine cytokine mRNA expression by qPCR or protein expression by ELISA (**E**). Activation of TBK1, IKKε, IRF3, ERK, p38 and JNK at various time points PI was analysed by immunoblot analysis. The phosphorylation levels of ERK, TBK1 and IRF3 (*n* = 3) were quantified using ImageJ (**F**). **G** WT and DUSP4 KO mice (*n* = 5) were infected with 5 × 10^4^ HSV-1 viruses through intravenous injection. Mice were monitored daily for survival (Log-rank test, *P* < 0.01). Unpaired *T*-test was used for statistical analysis. *P < 0.05; **P < 0.01. Data are representative of three independent experiments with similar results.

DUSP4 expression in WT BMDMs was found to be induced by various STING ligands including 2′3′-cGAMP, 3′3′-cGAMP and c-di-GMP (Fig. 3A). To confirm the regulatory role of DUSP4 in STING-mediated response, WT and KO BMDMs were stimulated with c-di-GMP. Increased expression of IFNα, IFNβ, IL-6 and TNFα at both mRNA and protein levels in KO cells compared to WT cells was detected (Fig. 3B, C). This increased expression of cytokines in KO cells was associated with increased activation of ERK1/2 (Fig. 3D), as well as TBK1 and IRF3. To validate the function of DUSP4 in STING-mediated immune response, WT and KO BMDMs were infected with HSV-1 viruses. Increased expression of IFNα, IFNβ, IL-6 and TNFα in KO cells compared to WT cells was detected (Fig. 3E). Furthermore, enhanced activation of TBK1 and IRF3, along with increased activation of ERK1/2 but not p38 or JNK (Fig. 3F), were observed in KO cells compared to WT cells. Furthermore, when infected with HSV-1 viruses, while all WT mice died around day 7 PI, the KO mice survived (Fig. 3G). As HSV-1 can also trigger the activation of RIG-I pathway [35, 36], these results suggest that DUSP4 suppresses both STING-mediated and RIG-I-mediated responses by inhibiting the activation of ERK1/2 and TBK1-IRF3 pathways.

### Increased STING-mediated immune response in DUSP4 KO mice was associated with enhanced susceptibility to ECM

**Fig. 4:**
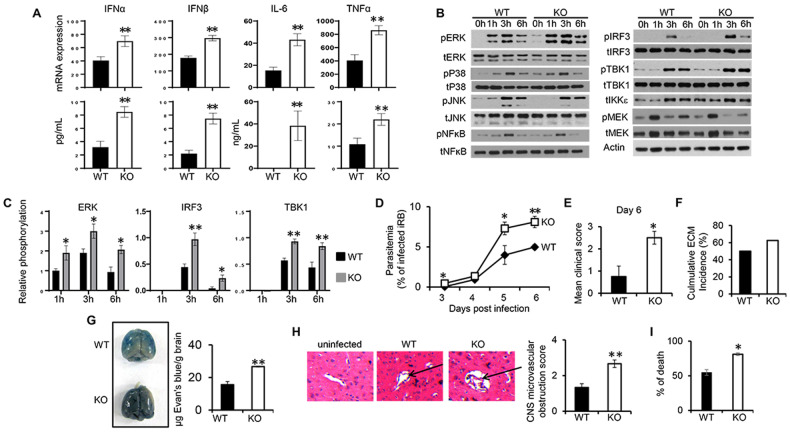
Increased susceptibility of DUSP4 deficient mice to experimental cerebral malaria. **A** WT and KO BMDMs were stimulated with DNA (250 ng/mL) isolated from *Plasmodium berghei ANKA* (*PbA*) for 6 h to examine the expression of *Ifnα, Ifnβ, Il6 and Tnfα* by qPCR. 24 h after stimulation, the concentration of IFNα, IFNβ, IL6 or TNFα in the supernatants was determined by ELISA. Activation of ERK, p38, TBK1 and IRF3 was analyzed by immunoblot (**B**) and the phosphorylation levels of ERK, TBK1 and IRF3 (*n* = 3) were quantified using ImageJ (**C**). **D-F** WT and DUSP4 KO mice (*n* = 5) were infected with 1 × 10^6^ of *PbA* infected red blood cells. Parasitemia was determined by flow cytometry (**D**). Experimental cerebral malaria (ECM) progression was monitored and recorded using a previous validated clinical scoring algorithm [53] (**E**). Cumulative incidence of ECM of WT (*n* = 14) and KO (*n* = 14) on day 7 PI from three independent experiments (**F**). **G** Qualitative (left) and quantitative (right) brain capillary permeability was assessed after intracardica perfusion of Evans blue-injected mice. **H** On day 7 post *PbA* infection, H&E-stained brain sections (left) and semi-quantitative score of brain microvascular obstruction are shown. Data are representative of three independent experiments with similar results. **I** Percentage of death of WT (*n* = 14) and KO (*n* = 14) mice from three independent experiments. Unpaired *T*-test was used for statistical analysis. *P < 0.05; **P < 0.01.

Type I IFNs induced by nonviral pathogens, such as intracellular parasite *Plasmodium falciparum*, may exacerbate the infection [16]. Based on the above results showing the negative regulatory role of DUSP4 in type I IFN expression, we aimed to determine whether DUSP4 may also play a role in malaria infection. Both WT and KO BMDMs were first stimulated with *Plasmodium berghei* ANKA (*Pb*A) DNA to assess the expression of type I IFNs and inflammatory cytokines. Increased expression of IFNα, IFNβ, IL-6 and TNFα in KO cells compared to WT cells was observed (Fig. 4A). This was in line with increased activation of ERK1/2 but not p38 or JNK (Fig. 4B, left and 4C), and increased activation of both TBK1 and IRF3 in KO cells compared to WT cells (Fig. 4B, right and 4C). Next, the physiological impact of the enhanced type I IFNs in the KO mice was tested using the experimental cerebral malaria model (ECM), wherein WT and KO mice were administrated with 1.0 × 10^6^ infected red blood cells (iRBC). Parasitemia was increased in KO mice on day 3, 5 and 6 PI compared to WT mice (Fig. 4D). Increased mean clinical score and increased ECM incidence were also observed in KO mice (Fig. 4E, F). In addition, assessment of the integrity of the blood–brain barrier (BBB) of the infected mice by Evans blue extravasation assay demonstrated increased BBB permeability in KO mice compared to WT mice (Fig. 4G). Furthermore, KO mice had increased brain microvascular obstruction and increased incidence of death compared to WT mice (Fig. 4H, I). Together, these results demonstrate that DUSP4 KO mice are more susceptible to *Pb*A infection.

### DUSP4 interacts with both TBK1 and IRF3, but only dephosphorylates TBK1

**Fig. 5:**
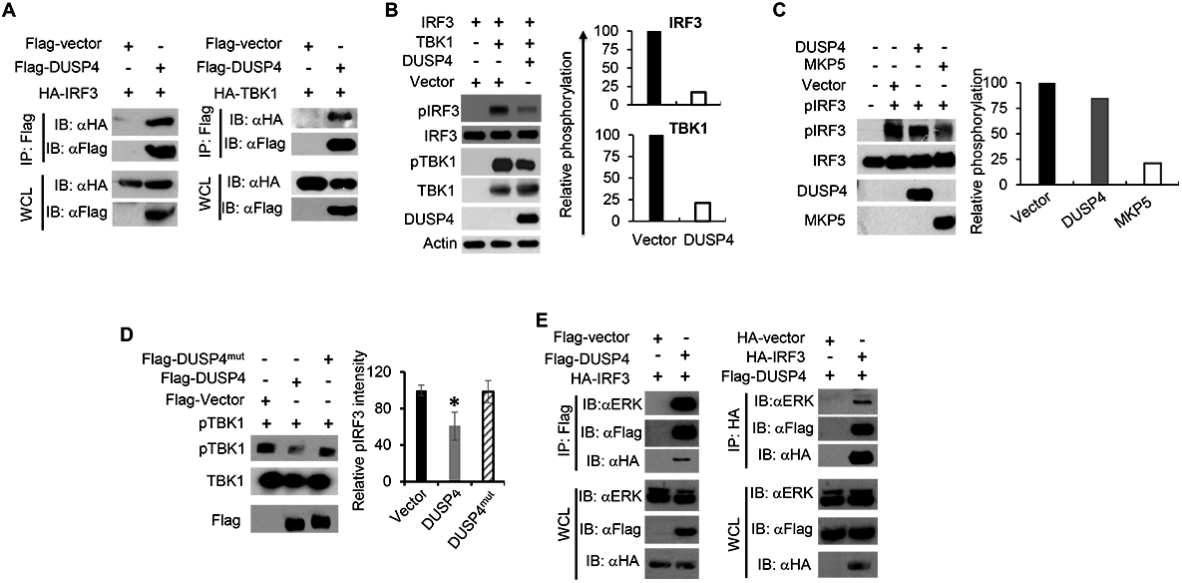
DUSP4 interacts with TBK1, IRF3 and ERK, and dephosphorylates TBK1. **A** HEK293T cells were transfected with flag-DUSP4 and HA-IRF3 or HA-TBK1. Interaction between DUSP4 and IRF3 (left) or TBK1 (right) was examined by immunoprecipitation (IP). **B** DUSP4 inhibits TBK1-mediated IRF3 phosphorylation. HEK293T cells were transfected with IRF3-expressing plasmids with or without TBK1- or DUSP4-expressing plasmids. Phosphorylation of IRF3 and TBK1, and total protein of IRF3, TBK1 and DUSP4, were determined by Immunoblot analysis. **C** Recombinant DUSP4 or MKP5 protein was incubated with phospho-IRF3 (pIRF3) to perform in vitro phosphatase assay. The level of IRF3 phosphorylation was analysed by immunoblot. **D** Purified pTBK1 was incubated with recombinant flag-DUSP4 or flag-DUSP4 phosphatase-dead mutant (flag-DUSP4^mut^) to perform in vitro phosphatase assay to determine the dephosphorylation of TBK1 by DUSP4. **E** HEK293T cells were transfected with HA-IRF3 and Flag-DUSP4 constructs in the combination indicated. Cell lysates were incubated with anti-Flag (left) or anti-HA (right) agarose beads to analyse the interaction between DUSP4, IRF3 and endogenous ERK by immunoblot. Data are representative of three independent experiments with similar results. Unpaired *T*-test was used for statistical analysis. *P < 0.05. WCL denotes whole cell lysate.

To elucidate the regulation of TBK1-IRF3 signalling by DUSP4, we examined the interaction between DUSP4 and TBK1, or IRF3. It was found that DUSP4 interacts with both IRF3 and TBK1 (Fig. 5A and Fig. S5), which is consistent with a previous finding [37]. To test the regulation of TBK1 and IRF3 activation by DUSP4, TBK1, IRF3 and DUSP4 expression plasmids were transfected into HEK293T cells. Consistent with previous studies [23, 26], over expression of TBK1 together with IRF3 resulted in phosphorylation of both TBK1 and IRF3 (Fig. 5B, lane 2). On the other hand, expression of DUSP4 suppressed the phosphorylation of both TBK1 and IRF3 (Fig. 5B, lane 3).

To test the possibility of dephosphorylation of TBK1 or IRF3 by DUSP4, we prepared DUSP4 recombinant protein, phosphorylated TBK1 (pTBK1) and pIRF3 to perform in vitro dephosphorylation assay [33]. We found that DUSP4 was unable to dephosphorylate pIRF3, unlike MKP5 which is known as an IRF3 phosphatase [33] (Fig. 5C). In contrast, the level of pTBK1 was significantly reduced by recombinant DUSP4, but not its phosphatase-dead mutant (DUSP4^mut^) (Fig. 5D), suggesting that DUSP4 inhibits TBK1 activation in a phosphatase-dependent manner. Detail characterisation of DUSP4-TBK1 interaction was also performed using various deletion mutant constructs of DUSP4 (Fig. S6A). It was found that deletion of a motif containing 84 amino acids located in front of the catalytic site of DUSP4 abolished its interaction with TBK1 (Fig. S6B). Together, these results demonstrate that DUSP4 might be a TBK1 phosphatase that can dephosphorylate TBK1 to suppress the activation of IRF3.

### DUSP4 forms a signalling complex with IRF3 and ERK

Although ERK is known to regulate the expression of type I IFNs [38], the underlying mechanism is still unclear. An examination of the interaction between DUSP4, IRF3 and ERK was carried out to determine the involvement of ERK in IRF3-type I IFN signalling. DUSP4 was shown to interact with both IRF3 and ERK (Fig. 5E, left), and IRF3 was able to interact with both ERK and DUSP4 (Fig. 5E, right). In addition, endogenous DUSP4 was found to interact with not only TBK1, but also IRF3 and ERK (Fig. S5). Together, these results suggest the presence of a signalling complex containing DUSP4, ERK, TBK1 and IRF3.

### DUSP4 regulates IFNβ expression through both ERK and TBK1-IRF3 signalling

**Fig. 6:**
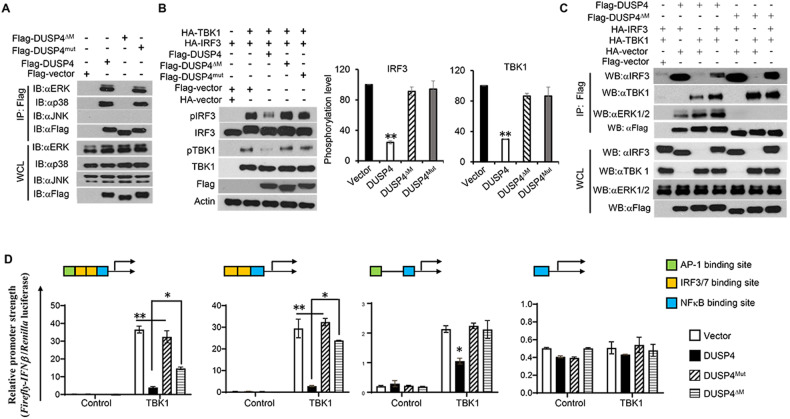
MAPK-interaction domain of DUSP4 is critical for its regulation of IRF3-type I IFN response. **A** Deletion of MAPK-interaction domain from DUSP4 abolished its interaction with ERK1/2 and p38. Flag-DUSP4, Flag-DUSP4^∆M^ or Flag-DUSP4^mut^ constructs were transfected to HEK293T cells to perform IP to examine their interaction with MAPKs including ERK1/2, p38 and JNK by immunoblot. **B** DUSP4 suppresses TBK1-mediated IRF3 activation, which is dependent on its MAPK-interaction domain. Immunoblot analysis of IRF3 phosphorylation in cells transfected with IRF3 together with TBK1 and DUSP4, DUSP4^ΔM^ or DUSP4^mut^. **C** Deletion of MAPK-interaction domain from DUSP4 resulted in its increased interaction with TBK1. Flag-DUSP4 or Flag-DUSP4^ΔM^ constructs were transfected to HEK293T cells together with indicated constructs perform IP to assess their interaction with IRF3 or TBK1. **D** IFNβ promoter luciferase construct containing one AP-1, two IRF3/7 and one NFκB binding site, or its deletion mutants lacking AP-1 binding site, the two IRF3/7 binding sites, or both, were transfected into HEK293T cells to examine their ability to suppress TBK1-mediated IFNβ promoter activity by dual-luciferase assays. Unpaired *T*-test was used for statistical analysis. *P < 0.05; **P < 0.01. Data are representative of three independent experiments with similar results.

DUSP4 interacts with ERK and p38 but not with JNK (Fig. 6A). Deletion of MAPK-interacting domain from DUSP4 (DUSP4^ΔM^ in Fig. S6A) abolished its interaction with ERK or p38, whereas its phosphatase-dead mutant (DUSP4^mut^) retained its interaction with both ERK and p38. To test whether the interaction between DUSP4 and MAPKs or its phosphatase activity would affect its regulation of TBK1 and IRF3, we transfected DUSP4, DUSP4^ΔM^ or DUSP4^mut^ together with TBK1 and IRF3 into HEK293T cells to assess the phosphorylation of IRF3. Interestingly, DUSP4, but neither DUSP4^ΔM^ nor DUSP4^mut^ is able to suppress the phosphorylation of both TBK1 and IRF3 (Fig. 6B), suggesting that both the phosphatase activity of DUSP4 and its interaction with ERK are important for its regulation of TBK1-mediated IRF3 activation.

Next, we tested if the interaction between DUSP4 and ERK interferes with the interaction between DUSP4 and TBK1 or IRF3. Consistent with the results in Fig. 5E, the interaction between DUSP4 and IRF3 was detected in an immunoprecipitation assay (Fig. 6C, lane 2). It was also able to interact with TBK1 (Fig. 6C, lane 3). Interestingly, deletion of MAPK-interacting domain from DUSP4 slightly increased its interaction with IRF3, but greatly increased its interaction with TBK1 (Fig. 6C, lane 5, 6 and 7). These results suggest that TBK1 may compete with MAPKs, mainly ERK, for interaction with DUSP4.

The IFNβ promoter region contains one AP-1 binding site, two IRF3/7 sites and one NFκB site [26]. To further elucidate the regulation of IFNβ expression by DUSP4, we cloned the IFNβ promoter region into pGL3 luciferase vector, and subsequently created mutant IFNβ promoter luciferase constructs with deletion of AP-1 binding site, IRF3/7 sites or NFκB site (Fig. 6D). Dual-luciferase assays were carried out after co-transfection of DUSP4 with various IFNβ promoter constructs together with or without TBK1. As expected, DUSP4 inhibits IFNβ transcriptional activity (Fig. 6D panel 1). Deletion of the AP-1 binding site resulted in a reduction in IFNβ promoter activity, and DUSP4 expression led to a further reduction (Fig. 6D, panel 2), demonstrating that DUSP4 inhibits AP-1-independent IFNβ expression. Similarly, deletion of IRF3/7 sites from the promoter also reduced its activity, which was further suppressed by DUSP4 (Fig. 6D, panel 3), indicating the importance of IRF3/7 in regulating IFNβ expression, and suggesting that DUSP4 can inhibit IRF3/7-independent IFNβ expression. On the other hand, deletion of both AP-1 and IRF3/7 sites resulted in very low promoter activity, and DUSP4 was no longer able to suppress it (Fig. 6D, panel 4). We also tested the ability of DUSP4^ΔM^ or DUSP4^mut^ in regulation of IFNβ transcriptional activity. It was found that the DUSP4^mut^ completely lost its ability in inhibition of IFNβ transcriptional activity. Interestingly, the DUSP4^ΔM^ was less able to suppress IFNβ transcriptional activity compared to WT DUSP4 (Fig. 6D, panel 1–2). Together, these results demonstrate that DUSP4 suppresses both AP-1- and IRF3/7-mediated IFNβ promoter activity, which is dependent on its phosphatase activity and partially on its interaction with MAPKs.

### IRF3 phosphorylation is regulated by ERK1/2

**Fig. 7:**
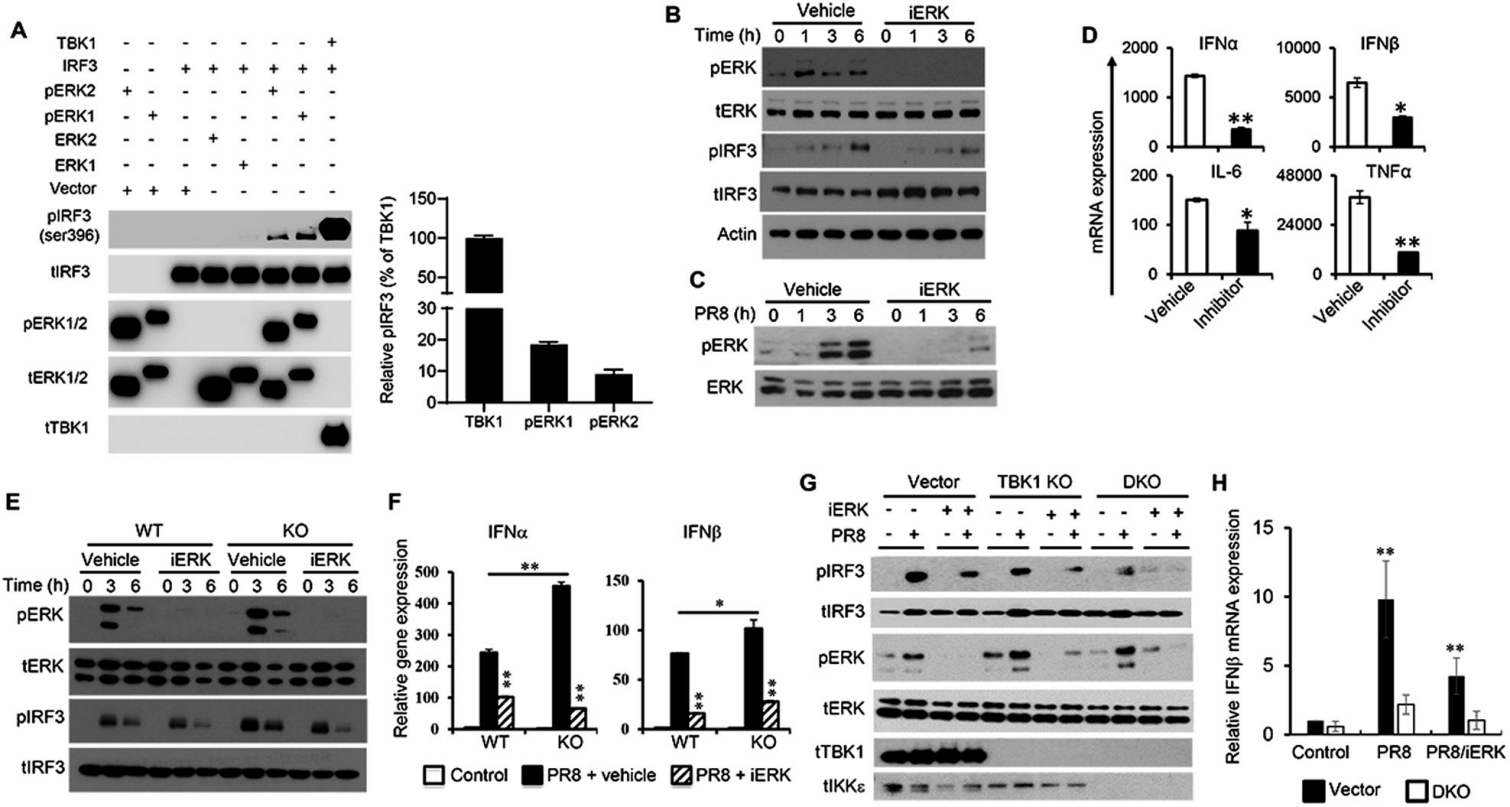
ERK1/2 regulate IRF3 activation important for type I IFN expression in response to influenza virus infection. **A** Recombinant IRF3 protein was incubated with purified pERK1, pERK2, ERK1, ERK2 or TBK1 for in vitro kinase assays. Phosphorylation of IRF3 was assessed by immunoblot and was quantified (*n* = 3) using ImageJ software. **B** BMDMs were pre-treated with vehicle or ERK inhibitor PD98059 (100 μM) for 1 h. Cells were then stimulated with 5’-ppp dsRNA (0.5 μg/mL) in the presence of vehicle or PD98059 respectively for the indicated period of time to assess the activation of ERK and IRF3 by immunoblot. **C**, **D** RAW264.7 cells were pre-treated with vehicle or ERK inhibitor PD98059 (100 μM) for 1 h followed by with PR8 at an MOI of 1. ERK activation at various time points PI was analysed by immunoblot (**C**). Cytokine expression with or without ERK inhibition was determined by qPCR. **E**, **F** WT and DUSP4 KO BMDMs were pre-treated with vehicle or PD98059 followed by infection with PR8 virus in the presence of vehicle or PD98059 for the indicated period of time to assess the activation of ERK and IRF3 by immunoblot (**E**). Expression of IFNα and IFNβ at 6 h PI was determined by qPCR (**F**). **G**, **H**. TBK1 deficient or TBK1 plus IKKε double deficient RAW264.7 cells were generated using CRISP/Cas9 technology. Cells were infected with PR8 virus with or without ERK inhibitor PD98059 to examine the phosphorylation of TBK1, IKKε, ERK, IRF3 by western blot analysis (**G**) or examine the expression of IFNβ by qPCR. Unpaired *T*-test was used for statistical analysis. *P < 0.05; **P < 0.01. Data are representative of two-three independent experiments with similar results.

To test the possible phosphorylation of IRF3 by ERK1/2, activated ERK1 and ERK2 were isolated and incubated with IRF3 to perform in vitro kinase assays to examine the phosphorylation of IRF3 at ser396. The results demonstrated that incubation of IRF3 with pERK1 or pERK2 resulted in the phosphorylation of IRF3 at ser396 residue, with stronger IRF3 phosphorylation by pERK1 than that by pERK2 (Fig. 7A, lane 7 and 6, respectively). However, compared to the TBK1-mediated IRF3 phosphorylation, ERK1/2-mediated phosphorylation was much weaker. Nevertheless, these results suggest the possibility of ERK1 and ERK2 as IRF3 kinases, with ERK1 having higher potency in activating IRF3 than ERK2.

To substantiate the regulation of IRF3 activation by ERK, BMDMs were stimulated with RIG-I ligand with or without treatment of PD98059 which inhibits ERK activation. RIG-I stimulation resulted in the activation of both ERK and IRF3, and PD98059 treatment abolished ERK1/2 activation and inhibited IRF3 phosphorylation (Fig. 7B).

To test the regulation of type I IFNs and proinflammatory cytokine expression by ERK, RAW264.7 cells were pre-treated with PD98059 followed by PR8 infection. We found that PD98059 successfully suppressed the activation of ERK1/2 in response to PR8 infection (Fig. 7C). Consequently, the expression of IFNα, IFNβ, IL-6 and TNFα was suppressed (Fig. 7D), suggesting that ERK activation is important for the expression of both type I IFNs and proinflammatory cytokines in response to influenza infection.

Subsequently, the level of ERK and IRF3 phosphorylation with or without PD98059 was examined in WT and DUSP4 KO BMDMs infected with PR8. PR8 infection induced the phosphorylation of both ERK and IRF3 at 3 and 6 h PI, and PD98059 treatment not only inhibited the phosphorylation of ERK, but also the phosphorylation of IRF3 in both WT and KO cells (Fig. 7E), suggesting that ERK regulates IRF3 activation in response to influenza virus infection. To further examine the regulation of ERK-mediated expression of type I IFNs by DUSP4, WT and KO BMDMs were infected with PR8 virus to examine the expression of IFNα and IFNβ with or without PD98059 treatment. Increased expression of both IFNα and IFNβ in KO cells compared to WT cells was observed (Fig. 7F). ERK inhibition resulted in a significant reduction of type I IFN expression in both WT and KO cells.

To substantiate the finding on the role of DUSP4 in the regulation of IRF3 activation and type I interferon expression, we generated TBK1 and IKKε double deficient RAW264.7 cells using CRISPR-Cas9 technology. Western blot analysis confirmed the successful generation of TBK1 deficient and TBK1 x IKKε double deficient (DKO) cells (Fig. 7G). In response to PR8 infection, reduced IRF3 phosphorylation in TBK1 KO cells compared to vector control cells was observed (Fig. 7G, lane 2 and 6). ERK inhibition reduced IRF3 phosphorylation in both types of cells (Fig. 7G, lane 4 and 8). Interestingly, a lower level of IRF3 phosphorylation in TBK1 x IKKε DKO cells (Fig. 7G, lane 10) compared to control or TBK1 KO cells was observed in response to PR8 infection, and ERK inhibition completely abolished this phosphorylation of IRF3 (Fig. 7G, lane 12). These results further supported the likelihood of IRF3 phosphorylation by ERK.

Next, we infected control and DKO cells with PR8 to examine IFNβ expression. A lower level of IFNβ expression in DKO cells compared to control cells was observed and ERK inhibition further reduced this expression (Fig. 7H). Together, these results demonstrated the important role of ERK in regulation of IRF3 activation and the expression of type I IFNs, which is suppressed by DUSP4.

## Discussion

In the battle against viral pathogens, the host employs various mechanisms to balance between the prompt and robust production of type I IFNs and restraining excessive production to avoid type I IFN-mediated immunopathologies [18]. On the other hand, viral pathogens utilize strategies to evade the type I IFN system, which are essential for their survival and spread to establish infection. Therefore, the identification of mechanisms that are used by both the host and the pathogens to modulate the type I IFN system will help to understand how this system operates to ensure the survival of the host or the microbe. This knowledge could inform the development of strategies to prevent and treat not only various infectious diseases but also type I IFN-mediated immunopathologies. Here, we identified DUSP4 as a common regulator in various PRR pathways, including RIG-I, TLR3, and STING, to modulate the production of type I IFNs. Specifically, we provide biochemical evidence showing that DUSP4 is a potential phosphatase of TBK1, but not IRF3, to inhibit IRF3 activation (Fig. 5C, D). In addition, we demonstrated the importance of ERK1/2 in the expression of type I IFNs in response to PRR stimulation and virus infection, possibly through phosphorylation of IRF3 (Fig. 7). Furthermore, we showed that DUSP4 is a component of a signalling complex, including TBK1, IRF3 and ERK, which is crucial for its regulation of type I IFN expression.

Regulatory function of MAPKs in the expression of proinflammatory cytokines, including IL-6 and TNFα, in infection was well established [39]. For instance, ERK1/2 activation was commonly observed upon PRR stimulation, contributing to the expression of proinflammatory cytokines. It has been shown that tumour progression locus 2 (Tpl2)-dependent activation of ERK1/2 in response to TLR4 stimulation was obligatory for the expression of TNFα [40]. It is also known that activation of MAPKs induces AP-1 transcriptional factor, which, together with IRF3 and NFκB, to regulate the expression of type I IFNs [41]. However, how individual MAPK, including ERK1 and ERK2, regulates the expression of type I IFN is unclear. Accumulating evidence demonstrates the importance of ERK1/2 in the regulation of type I IFNs. For example, deficiency of Tpl2 blocked the activation of ERK1/2 in response to TLR4 and TLR9 stimulation, leading to an increased expression of IFNβ in BMDMs [42]. This was attributed to a reduced expression of ERK-regulated *c-fos*, suggesting that ERK1/2 may negatively regulate type I IFN expression. On the other hand, other studies showed that ERK1/2 are required for the expression of type I IFNs in response to virus infection. For instance, in response to myxoma virus infection, inhibition of ERK1/2 activation suppressed type I IFN expression, consequently rendering non-permissive cells highly permissive to myxoma virus infection [38]. This was due to a complete blockade of myxoma virus-induced IRF3 activation by ERK1/2 inhibition, resulting in an impairment in type I IFN expression. Interestingly, activated ERK1/2 induced by myxoma virus failed to translocate into the nucleus, indicating that ERK1/2 may regulate the activation of IRF3 in the cytoplasm. However, whether ERK1/2 directly or indirectly regulate the activation of IRF3 upon myxoma infection was unclear. Here we showed that ERK1/2 are able to interact with and possibly phosphorylate IRF3 (Figs. 5E and  7A), thereby positively regulating the expression of type I IFNs in response to virus infection. Our results therefore provide evidence to support ERK1 and ERK2 as possible IRF3 kinases, directly regulating the IRF3-type I IFN response. Of note, our findings do not exclude the possibility of ERK1/2 in regulation of type I IFN expression through AP-1 or other molecules. We observed that DUSP4 was able to suppress both AP-1- and IRF3/7-mediated IFNβ transcriptional activity (Fig. 6D). It is possible that there are two pools of activated ERK1/2, one of which contributes to the expression of type I IFNs through IRF3, and the other through AP-1.

In addition, the differences on the roles of ERK1/2 in innate immunity observed in the aforementioned studies [38, 42] and ours could be attributed to the recruitment of ERK1/2 to different signalling complexes under various physiological conditions. It is possible that TLR4- and TLR9-stimulated ERK1/2 are preferentially recruited to a signalling complex leading to the expression of *c-fos*, whereas virus-stimulated or a fraction of virus-stimulated ERK1/2 is recruited into the TBK1-IRF3 complex to regulate type I IFN expression. Such recruitments could be mediated by proteins such as DUSP4 or scaffold proteins which play important roles in cell signalling by directing components of a specific pathway to the correct cellular location, linking them to a multi-enzyme complex, and facilitating their functional interaction [43].

Interestingly, deletion of ERK1/2 binding site from DUSP4 increased its interaction with TBK1 (Fig. 6C), suggesting that the interaction of DUSP4 with ERK1/2 reduces its interaction with TBK1, and without MAPK-interaction domain, DUSP4 could function as a scaffold protein for TBK1 and IRF3 to facilitate IRF3 activation. As such, when the MAPK-interaction domain is deleted, more TBK1 is recruited to DUSP4-IRF3, resulting in more IRF3 phosphorylation. This could explain why there was no dephosphorylation of TBK1 and IRF3 observed when DUSP4^ΔM^ was overexpressed (Fig. 6B). Interestingly, DUSP4^ΔM^ was still able to inhibit IFNβ transcriptional activity (Fig. 6D). The different results between the two experiments could be due to the different sensitivity of the two assays. Although both ERK1 and ERK2 were able to phosphorylate IRF3 (Fig. 7A), the phosphorylation efficiency was much lower compared to that of TBK1. Therefore, it is possible that DUSP4 may indirectly regulate TBK1-mediated IRF3 activation through interaction with ERK which reduces the recruitment of TBK1 to the signalling complex. It is possible that the primary substrate of DUSP4 is ERK1/2. However, in a context where DUSP4 is abundantly expressed, ERK1/2 activation is weakly induced, or it is recruited to a signalling complex where ERK1/2 is not present or present at a low concentration, it will then be able to target TBK1. Such a scenario may apply to other DUSPs to explain their different preferences for substrates in different types of cells, tissues or in response to various stimuli. Such flexibility of substrate preference is perhaps an intrinsic feature of many phosphatases, providing that there are over 500 kinases but only about 189 known phosphatases in humans [44, 45], so that the smaller number of phosphatases are able to counteract the action of a much larger number of kinases.

The expression of DUSP4 in PBMCs from influenza patients with mild symptom was increased, whereas in those from patients with severe symptom, it was significantly reduced (Fig. 1A). It is possible that in patients with mild symptom, influenza infection increased the expression of DUSP4, providing a safeguard for the expression of type I IFNs and the degree of immune activation. This ensured the control and eradication of the viruses without causing excessive immune-mediated collateral damage. In individuals who developed severe symptom, the reduced expression of DUSP4 caused by either host genetic reason or viral manipulation, might contribute to the overabundant production of both proinflammatory cytokines and type I IFNs, leading to uncontrolled immune activation and, therefore, the development of severe symptom. However, further study will be required to define the exact mechanism underlying this phenomenon.

In summary, we identified DUSP4 as an important modulator in innate immune signalling, constraining the expression of both type I IFNs and inflammatory cytokines in response to infection, likely through TBK1 and ERK1/2 (Fig. S7). Our study suggests that it situates at converging points of various PRR pathways to regulate the strength of the ensuing response, influencing the outcomes of microbial infection. Furthermore, our study also provides evidence of ERK1/2 as potential IRF3 kinases and sheds light on the regulation of the IRF3-type I IFN system.

## Materials and methods

### Mice, viruses and parasites

DUSP4 KO (*dusp4*^*tm1a/tm1a*^) mice were obtained from Wellcome Trust Sanger Institute, and were crossed with C57BL/6 mice for 12 generations. Age- and sex-matched groups were used for the experiments. All murine experiments were performed in accordance with guidelines from the Singapore National Advisory Committee on Laboratory Animal Research. The protocol was reviewed and approved by the National University of Singapore Institutional Animal Care and Use Committee (identification number: 2013-05890). WT and DUSP4 knockout mice were randomly assigned to experimental groups.

The influenza virus A H1N1 strain A/Puerto Rico/8/34 (PR8) was propagated by allantoic inoculation of 10-day embryonated chicken eggs. Virus titers were determined as plaque-forming units (PFU) on MDCK monolayers by plaque assay. Mice were infected with 50 or 500 PFU of PR8 through intranasal inoculation. Following infection, viral titers in the lungs at various time points post-infection (PI) were determined. Bronchoalveolar lavage (BAL) fluids from the mice were used to determine cytokine production. Total RNA from the lungs was extracted to determine mRNA expression of different cytokines or viral genes by quantitative RT-PCR (qPCR).

Herpes Simplex Virus-1 (HSV-1) was propagated on Vero cells. Virus titers were determined as 50% tissue culture infective dose (TCID50) on Vero cells by TCID50 assay.

*Plasmodium berghei* ANKA (*Pb*A) was a generous gift from Professor Laurent RÉNIA (SIgN, Singapore). Blood-stage parasites for experimental infection were obtained from donor mice at 6-10 days after inoculation with frozen stock. Mice were infected with 1.0 × 10^6^ parasitized red blood cells through intraperitoneal injection. Following infection, survival and parasitemia were monitored throughout the observation period. Clinical experimental cerebral malaria (ECM) scores were assessed as previously described [46] by the presentation of the following signs: ruffled fur, hunching, wobbly gait, limb paralysis, convulsions, and coma. Each sign was given a score of 1. Animals with severe ECM (accumulative scores ≥ 4) were sacrificed by CO_2_ asphyxiation according to ethics guidelines, and the day of death was deemed to be the following day.

### Cell culture, stimulation, infection and transfection

Bone marrow cells were flushed out from the femurs and tibias of the mice with sterile phosphate-buffered saline (PBS) to prepare bone marrow derived macrophages (BMDMs). Red blood cells were lysed using ammonium-chloride- potassium lysis buffer. The cells were cultured in complete RPMI 1640 media (Hyclone) supplemented with 10% (v/v) fetal bovine serum (FBS) (Sigma-Aldrich), 1 μg/mL Penicillin, 1 μg/mL Streptomycin and 20 ng/mL of macrophage colony-stimulating factor (M-CSF) (PeproTech) in a humidified 37^o^C incubator with 5% CO_2_. On day 2, 4 and 6, fresh RPMI 1640 medium supplemented with 20 ng/mL M-CSF was added to the culture. On day 7, the macrophages were harvested for experiments.

RAW264.7 cells were maintained in RPMI 1640 medium supplemented with 10% (v/v) FBS. Cells at 50% confluence in six‐well plates were transfected with 2–3 μg plasmid using Lipofectamine LTX (Invitrogen) according to the manufacturer’s instructions [33].

HEK293T cells, Vero cells and MDCK cells were cultured in Dulbecco’s Modified Eagle’s Medium (DMEM) (Hyclone) supplemented with 10% (v/v) FBS at 37 °C incubator with 5% CO_2_. HEK293T cells were transfected at 70-80% confluency with Mirus bio LT1 or Mirus bio IT 293 (Mirus Bio) according to the manufacturer’s instructions.

To study cellular response to pattern recognition receptor (PRR) stimulation, cells were transfected with 0.5 μg poly(I:C) (Sigma-Aldrich), 0.5 μg 5′ppp dsRNA (InvivoGen), 0.5 μg 2′-3′ cGAMP (InvivoGen), 0.5 μg 3′-3′ cGAMP (InvivoGen), or 0.5 μg c-di-GMP (InvivoGen) using Lipofectamine LTX (Invitrogen) according to the manufacturer’s instructions and were harvested at various time-points post transfection for analysis. For cellular response to virus infection, cells were infected with PR8 (MOI: 1) or HSV-1 (MOI: 0.01). Cells were harvested at various time-points PI for examination.

For ERK inhibition assays, BMDMs were pre-treated with ERK inhibitor PD98059 (100 µM) (Sigma-Aldrich), or DMSO (Sigma-Aldrich) as vehicle control for 1 h. After pre-treatment, the cells were infected with PR8 (MOI: 1) in the presence of PD98059 (100 µM) or DMSO for the indicated time-points before harvesting the samples for ELISA or qPCR analysis.

### Plaque assay

Lungs were taken from mice infected and homogenised to determine viral load using a modified plaque assay [47]. Briefly, monolayer MDCK cells were seeded in 24-well plates. The following day, homogenised lung tissue samples or virus stocks were serially diluted in serum-free Eagle’s minimum essential medium (EMEM) (Lonza) containing 1 µg/mL TPCK-treated trypsin (Sigma-Aldrich). Two hundred fifty microliters diluted sample was added into each separate well and incubated at 37 °C in 5% CO_2_ for 1 h. The infected cells were washed three times with PBS before a 1.2% Avicel (FMC Biopolymer) in EMEM overlay was added to each well. The plates were incubated at 37 °C in 5% CO_2_ for 60–70 h before fixation with 8% formaldehyde (Merck) and staining with crystal violet dye (Sigma-Aldrich). The lowest dilution with distinct plaque formation was counted to determine the viral loads in the lungs of the mice or determine the virus titers in the stock.

### Measurement parasitemia by FACS

Parasitemia in *Pb*A infected mice was determined by measuring the percentage of parasitized red blood cells using a tri-colour method (TCM) [48]. Briefly, 1 μL of whole blood cells was added to a tube containing 100 μL of PBS. Dihydroethidium (Sigma-Aldrich), Hoechst 33342 (Sigma-Aldrich), and allophycocyanine (APC)-conjugated anti-CD45.2 were added to the blood sample, followed by incubation for 20 min at room temperature in the dark. Cells were subsequently analysed with the BD FACSCalibur (BD Bioscience), using the CellQuest^TM^ Pro software.

### Assessment of brain vascular permeability

BBB permeability during *Pb*A infection was assessed using the Evans blue assay as described previously [49]. Briefly, mice were injected intravenously (i.v.) with 200 μL of PBS–2% Evans blue (Sigma-Aldrich), sacrificed 1 h later and perfused intracardially with PBS. Brains were surgically removed, weighed, and placed in 2 mL of 100% formamide (Sigma-Aldrich) for 48 h at 37 °C to extract the Evans blue dye from the tissue. Absorbance was subsequently measured at 620 nm using a Bio-Rad spectrophotometer. Evans blue concentrations per gram of brain tissue were calculated from a standard curve prepared with a known concentration of Evans blue in formamide.

### Immunofluorescence and confocal microscopy

BMDMs grown on glass coverslips were infected with PR8 virus at an MOI of 2 for 6 h. Cells were fixed with 3.7% formaldehyde for 15 min at 37 °C. Fixed cells were washed twice with PBS, twice with NH_4_Cl (4 mM) in PBS, twice with PBS, followed by permeabilization with 0.2% Triton X‐100 (Bio-Rad) in PBS for 15 min at room temperature. Blocking was carried out with 2% bovine serum albumin and 7% fetal bovine serum in PBS for 60 minutes at room temperature. Cells were then incubated with rabbit anti‐IRF3 antibody (Santa Cruz) in blocking solution overnight. Samples were washed three times with 0.1% Triton X‐100‐containing PBS before incubation with Alexa Fluor 555‐conjugated donkey anti‐rabbit IgG for 60 minutes, washed three times with 0.1% Triton X‐100‐containing PBS, and incubated with DAPI for 5 min, washed twice and mounted using Fluorsave. Confocal fluorescence images were captured on Zeiss LSM510 META microscope.

### Plasmid construction, RNA extraction, reverse transcription and real-time PCR

Mouse DUSP4 and IRF3 cDNA were generated by PCR using a mouse cDNA library with respective primers. Mutant constructs were generated from full-length cDNA constructs with suitable primers and cloned into mammalian expression vectors with either Flag tag or HA tag.

RNA samples from cell or homogenised tissues were extracted using Trizol® (Invitrogen) to synthesize cDNA with 1 μg of RNA using the oligo-dT primers (Promega) and the Improm-II Reverse transcription kit (Promega) according to the manufacturer’s instruction. qPCR was conducted using Fast-SYBR® Green Master Mix (ABI Applied Biosystem) to assess the expression of various genes. To normalize of the amount of cDNA between samples, Glyceraldehyde 3-phosphate dehydrogenase (GAPDH) was used as an endogenous control. Relative gene expression levels were calculated using 2^−∆∆Ct^ method [50]. The data of WT and KO samples were normalized to respective unstimulated value of WT sample. Specificity of amplification reactions was confirmed through melting curve analysis to validate the primer pair specificity.

qPCR primers used in this study:

Murine *Ifnb1* forward: 5′-CCCTATGGAGATGACGGAGA-3′;

Murine *Ifnb1* reverse: 5′-CTGTCTGCTGGTGGAGTTCA-3′;

Murine *Ifna* forward: 5′-AGGCTCAAGCCATCCCTGT-3′;

Murine *Ifna* reverse: 5′-CAGGGGCTGTGTTTCTTCTC-3′;

Murine *Il6* forward: 5′-GATGCTACCAAACTGGATATAATC-3′;

Murine *Il6* reverse: 5′-GGTCCTTAGCCACTCCTTCTGTG-3’;

Murine *Tnfa* forward: 5′-TCCCAGGTTCTCTTCAAGGGA-3′;

Murine *Tnfa* reverse: 5′-GGTGAGGAGCACGTAGTCGG-3′;

Murine *Rantes* forward: 5′-ATATGGCTCGGACACCA-3′;

Murine *Rantes* reverse: 5′-ACACACTTGGCGGTTCCT-3′;

Murine *Gapdh* forward: 5′-GAGAACTTTGGCATTGTGG-3′;

Murine *Gapdh* reverse: 5′-ATGCAGGGATGATGTTCTG-3′;

PR8 HA forward: 5′-AGTGCCCAAAATACGTCAGG-3′;

PR8 HA reverse: 5′-TCCCGTTAATGGCATTTTGT-3′;

PR8 NA forward: 5′-CCTGATACCGGCAAAGTGAT-3′;

PR8 NA reverse: 5′-ACTCCGTTTGCTCCATCAAC-3′.

### Co-immunoprecipitation and immunoblot analysis

HEK293T cells were lysed in 250 μL NP40 lysis buffer (50 mM Tris‐HCl; pH 7.3, 150 mM NaCl, and 1% (v/v) NP40) along with freshly added protease and phosphatase inhibitors (Roche). Lysates were incubated at 4°C overnight with anti‐Flag antibody conjugated to agarose beads (Sigma-Aldrich) or anti-HA antibody conjugated to agarose beads. The beads were washed with lysis buffer and analysed by western blotting. Primary antibodies used were polyclonal anti‐Flag (Sigma-Aldrich), anti‐HA (Zymed), anti‐ERK1/2, anti-IRF3, or anti-TBK1 (Cell Signaling Technology). Donkey anti-rabbit HRP-conjugated antibody (Amersham biosciences) was used as the secondary antibody.

Cell lysates were prepared as previously described [51], resolved in a 10% or 12% Tris/Glycine gel (10% or 12% acrylamide, 1.5 M Tris (pH 8.8), 10% SDS, 10% APS, Tetramethylethylenediamine), and were subjected to western blot analysis with anti-pERK, anti-pp38 and anti-pJNK antibodies (Cell Signaling Technology) for MAP kinases activation, anti-pIRF3 (Ser396) (Cell Signaling Technology) for IRF3 phosphorylation, or anti-pTBK1, anti-TBK1 or anti-IKKε for their phosphorylation and expression, respectively. The blots were exposed to Amersham^®^ Hyperfilm® ECL™ and MP Autoradiography Films (GE Healthcare). The intensity of the specific bands was quantified using ImageJ software.

### In vitro phosphatase assay and kinase assay

For phosphatase assay, cell lysates from HEK293T cells expressing Flag-vector, Flag-DUSP4, Flag-DUSP4^mut^ (C296S), or HA-IRF3, HA-TBK1 were incubated with anti-Flag or anti-HA conjugated to agarose beads respectively for 3 h at 4 °C to purify recombinant protein. To prepare pIRF3 or pTBK1, RAW264.7 macrophages transfected with HA-IRF3 or HA-TBK1 were treated with 100 ng/mL of lipopolysaccharide (Sigma-Aldrich) to induce TBK1 and IRF3 phosphorylation. Cells were harvested and treated with lysis buffer containing protease and phosphatase inhibitors (Roche), and pIRF3 or pTBK1 was purified by anti-HA agarose beads. In vitro dephosphorylation assay was performed by incubating equal volumes of purified pIRF3 or pTBK1 with recombinant Flag-vector, Flag-DUSP4, Flag-DUSP4^mut^, or Flag-MKP5 for 2 h at 37 °C in phosphatase assay buffer as described previously [52].

For kinase assay, 293T cells were transfected with HA-tagged ERK1, ERK2, IRF3, TBK1, or Flag-tagged RSK. Cells transfected with ERK1 or ERK2 were treated with 100 ng/mL EGF (Sigma–Aldrich) for 10 minutes prior to harvest to enhance their activation and phosphorylation. Cell lysates were first mixed with anti-HA agarose beads (Thermo scientific) or anti-FLAG® M2 Magnetic Beads (Sigma-Aldrich) for 3 h at 4 °C to prepare recombinant IRF3, TBK1 and RSK, or pERK1 and pERK2. Beads were then washed twice with lysis buffer and twice with PBS. Phosphorylation reactions were performed using 80 μL of kinase assay buffer (Cell Signalling Technology) supplemented with ATP at a final concentration of 200 μm, followed by incubation at 30 °C for 30 min. Reactions were terminated with 6× SDS loading buffer, and proteins were subjected to SDS-PAGE and western blot analysis.

### Enzyme-linked immunosorbent assay (ELISA)

TNFα and IL-6 cytokines in the cell culture supernatants or BALs was determined by sandwich ELISA using 96-well MaxiSorp^TM^ Immunoplates (Nunc). Capture and detection antibodies were purchased from BD Pharmingen. Streptavidin-HRP was purchased from Biolegend. O-phenylenediamine dihydrochloride (Sigma-Aldrich) solution was prepared according to the manufacturer’s protocol. IFNα and IFNβ ELISA kits (PBL Interferon Source) were used for the measurement of IFNα and IFNβ concentrations according to the manufacturer’s protocol. Concentrations of these cytokines were determined by reading the absorbance at 450 nm using the BioTek^®^ Microplate Reader (BioTek).

### Dual-luciferase assay

HEK293T cells seeded on 24-well plates were transfected with 400 ng/mL of IFNβ promoter together with various gene expressing plasmids. As an internal control, 10 ng pRL-TK Renilla was transfected simultaneously. Eighteen hours after transfection, the cells were washed with PBS and lysed with passive lysis buffer (Promega) for 1 h at room temperature. Luciferase activity was then measured in whole cell lysates using the Dual-Luciferase Assay System (Promega) according to the manufacturer’s instruction.

### Statistical analysis

Data are presented as mean ± SEM. Statistical significance of the differences between WT and KO groups was determined by 2-tailed unpaired student *t* test with GraphPad Prism (*P < 0.05, and **P < 0.01). Mann–Whitney non-parametric test was use to analyse the differences between healthy control and mild/severe influenza patients. Log-rank test was used to analyse WT and KO mice survival in response to virus infection.

### Supplementary information

Supplemental Figures

Supplemental files

Table S1

Reproducibility checklist

## Data Availability

The manuscript does not contain RNA-Seq or other large data sets. Detailed information on antibodies used in the manuscript is provided in the supplemental table. Raw western blots are available in the Supplemental file.
