# Supplementary material for: DUSP4 modulates RIG-I- and STING-mediated IRF3-type I IFN response
Source: Cell Death Differ. 2024 Feb 21;31(3):280–91. doi: 10.1038/s41418-024-01269-7 (PMC10923883; doi:10.1038/s41418-024-01269-7)
Supplement: Supplementary file 1 — Supplemental Figures [file 41418_2024_1269_MOESM1_ESM.pdf]

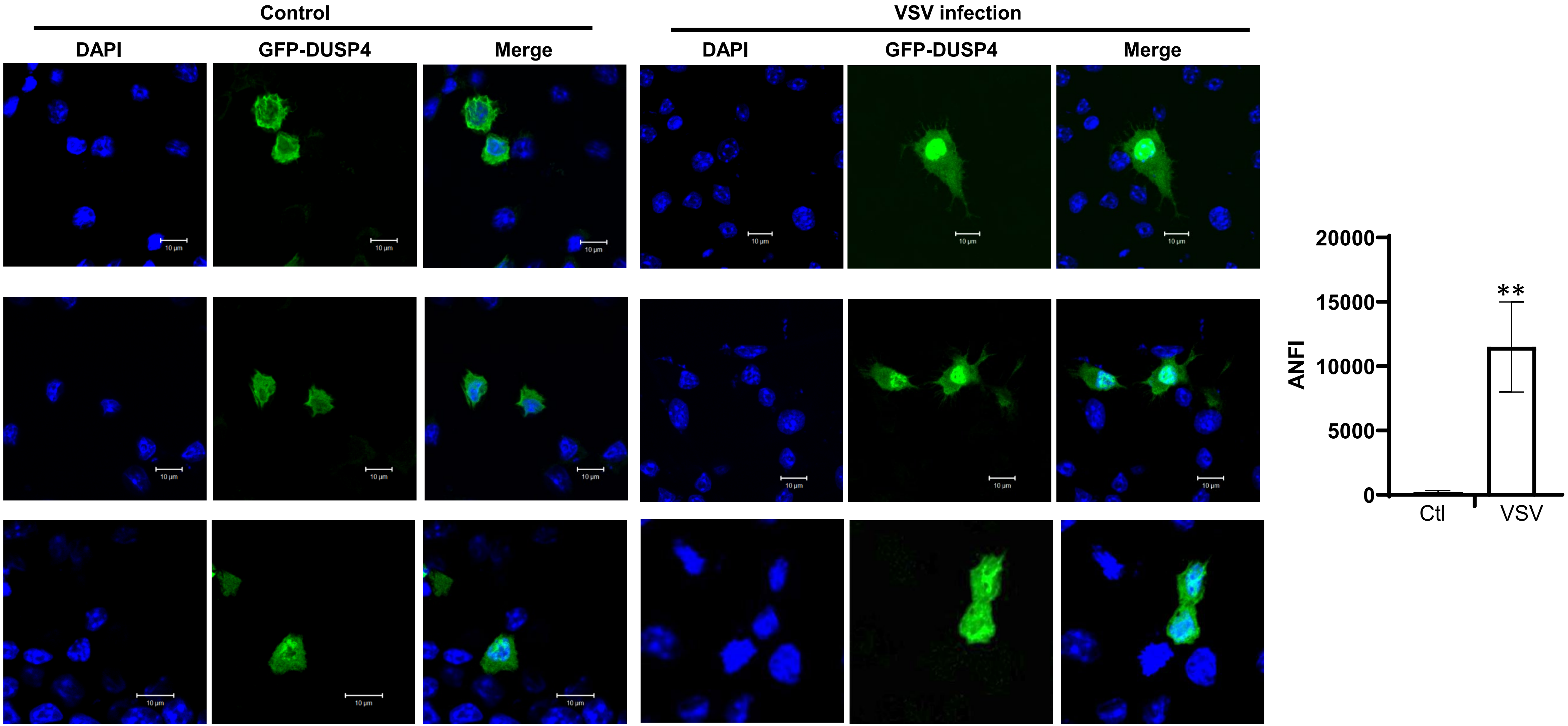

Figure S1 VSV infection leads to nuclei accumulation of DUSP4. RAW264.7 cells were transfected with GFP-DUSP4 constructs. After overnight resting, cells with or without VSV infection for 6 hours were subjected confocal microscopy examination for the subcellular localization of DUSP4. Cell nuclei were stained with DAPI. Results presented in each row represent data from one experiment. Nuclear fluorescent intensity of twenty cells from each experiment was measured using Image J. and the average intensity from three experiments was used to determine average nuclear fluorescent intensity (ANFI) in control (Ctl) and VSV infected cells.

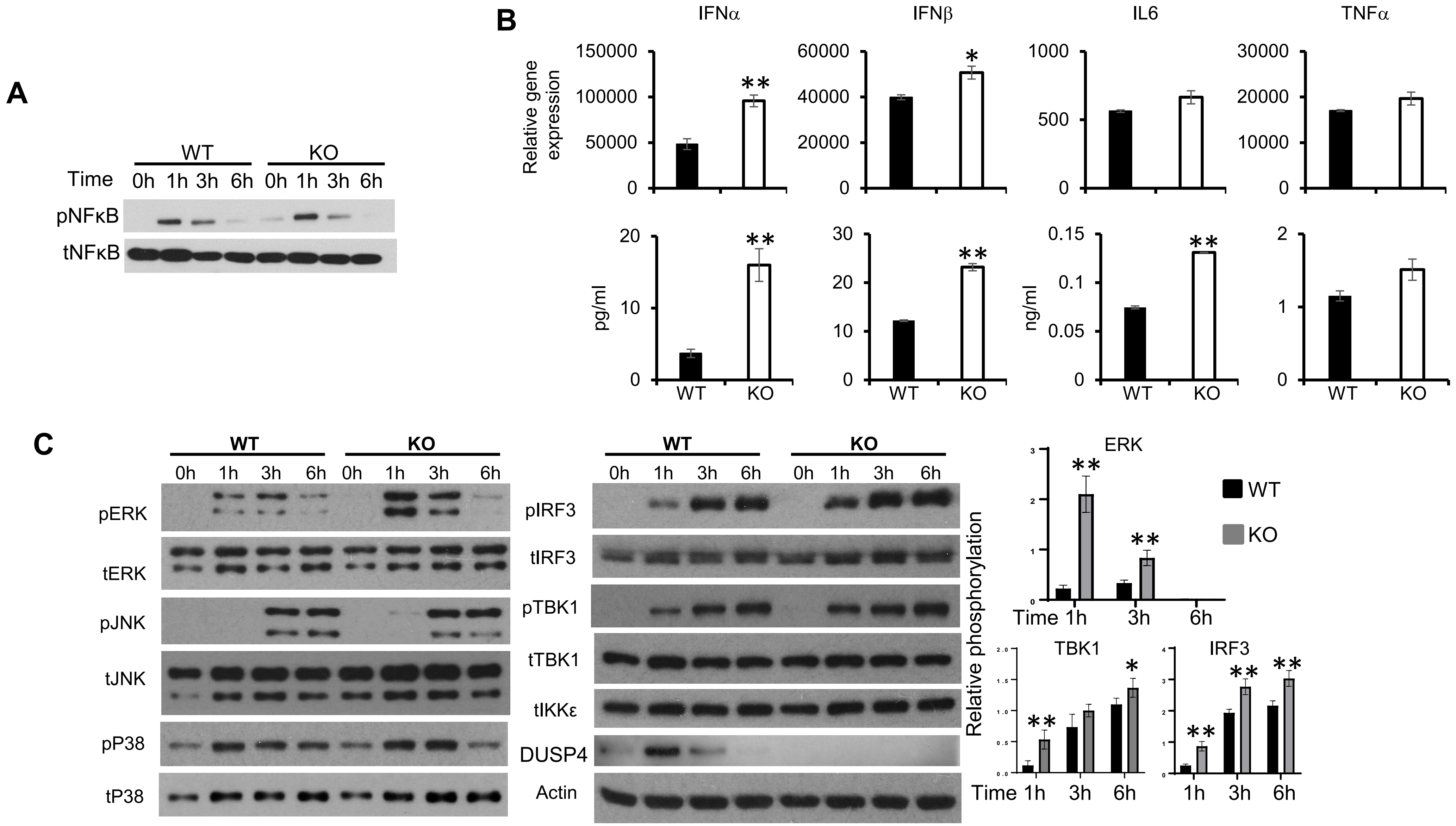

Figure S2 Increased cytokine expression, ERK and TBK1-IRF3 activation in DUSP4 KO BMDMs in response to TLR3 activation.
(A) Comparable NFκB activation between WT and DUSP4 KO BMDMs in response to 5'-PPP dsRNA (0.5 μg/mL) stimulation (B) WT and DUSP4 KO BMDMs were stimulated with poly I:C (0.5 μg/mL). mRNA expression of IFNα, IFNβ, IL-6 and TNFα at 6 h after stimulation was examined by qPCR. Protein expression of type I IFNs at 6 h and that of IL-6 and TNFα at 24 h P.I. were determined by ELISA. (C) Activation of ERK, JNK, p38, IRF3, TBK1 and IKKε and the expression of DUSP4 at the indicated time points post stimulation was analyzed by immunoblot. The phosphorylation levels of ERK, TBK1 and IRF3 (n=3) were quantified by ImageJ.

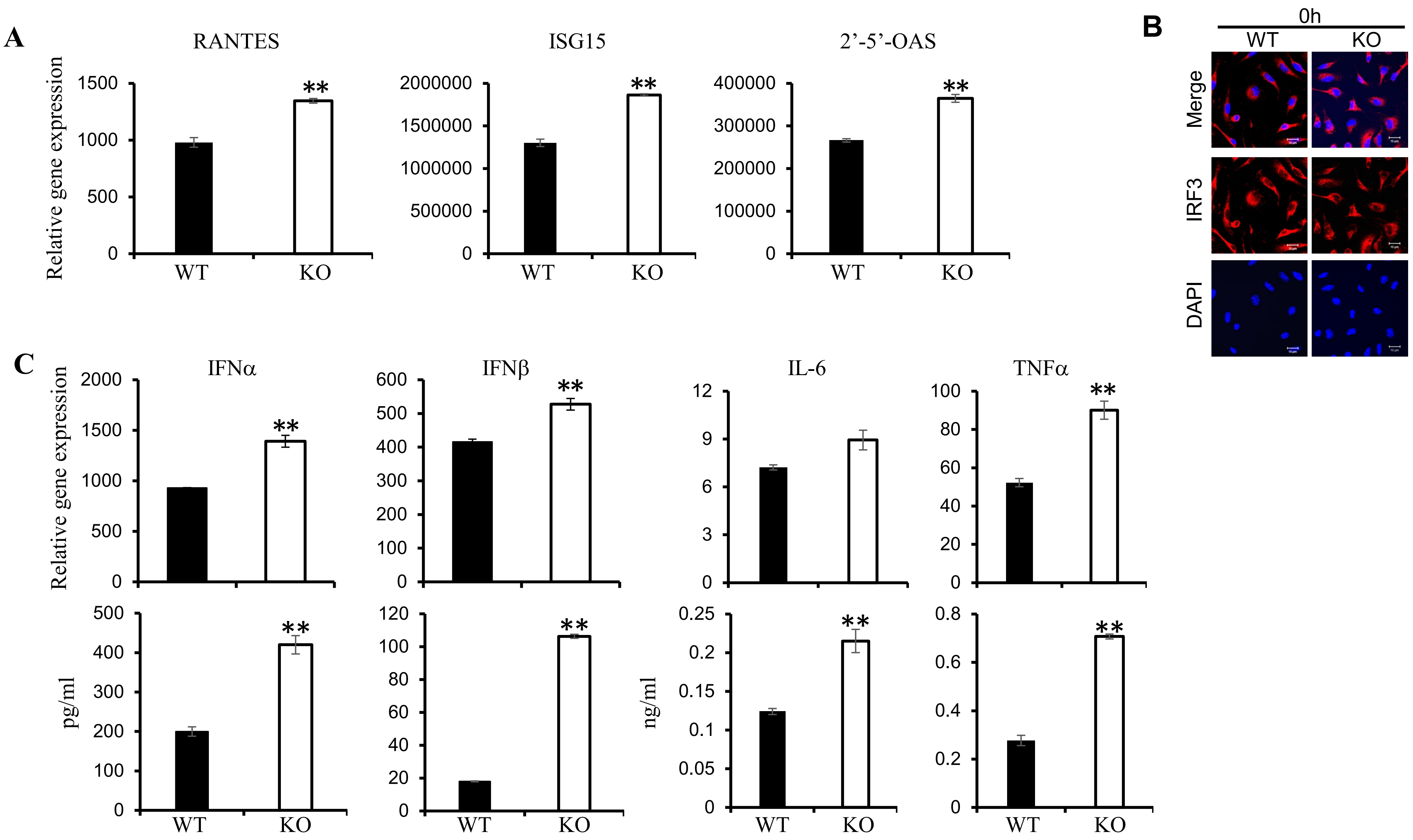

Figure S3 Increased type I IFN response in DUSP4 KO BMDMs and BMDCs in response to influenza infection. (A) WT and DUSP4 KO BMDMs were infected with PR8 (MOI, 1). Expression of the indicated ISGs at 6 h P.I. was analyzed by qPCR. (B) Confocal microscopy of WT and KO BMDMs without stimulation showing comparable IRF3 nuclear accumulation between WT and DUSP4 KO BMDMs. (C) WT and KO BMDCs were infected with PR8 virus (MOI, 1) for 6 h to examine the expression of IFNα, IFNβ, IL-6 and TNFα using qPCR and ELISA.

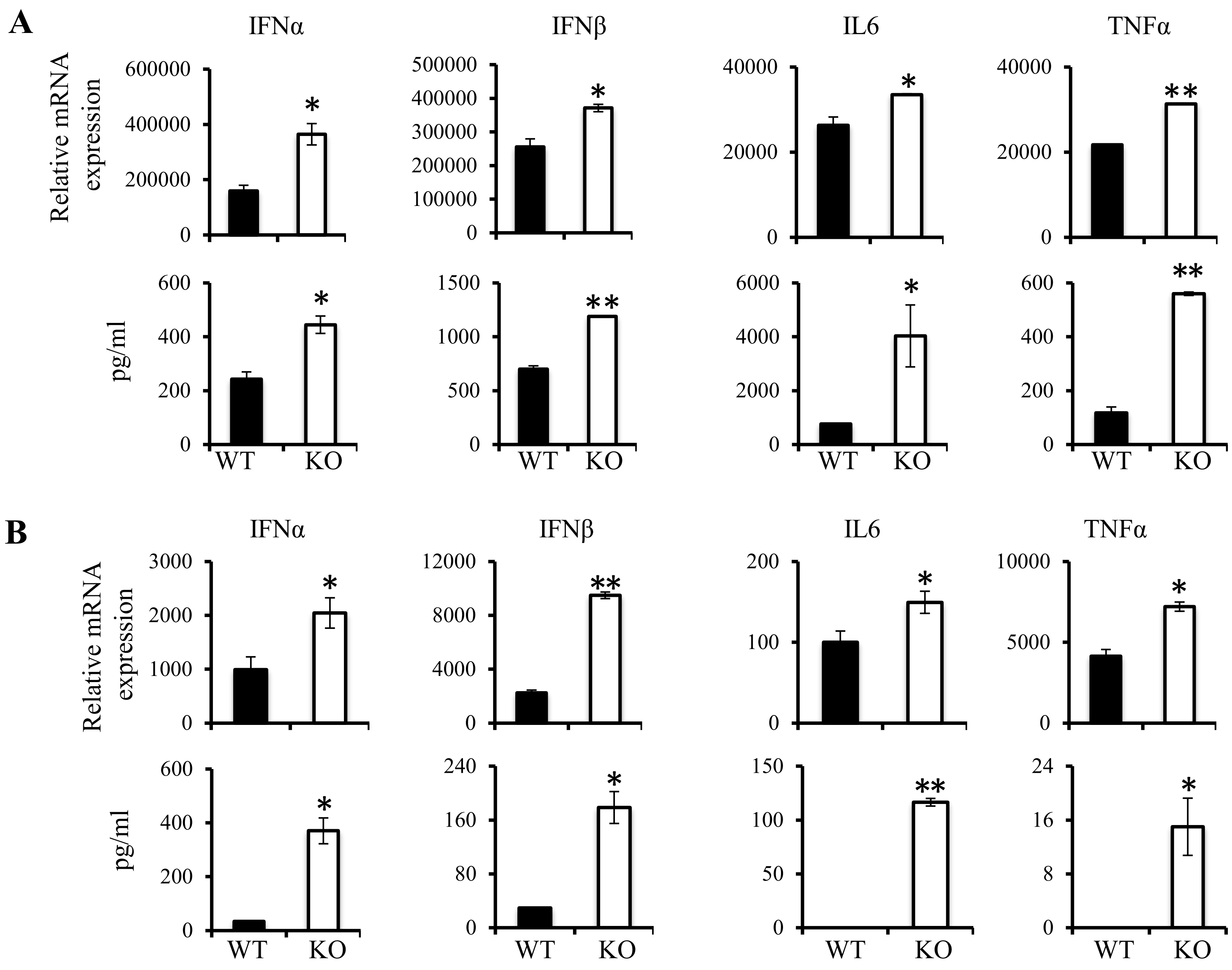

Figure S4 Increased type I IFNs and pro-inflammatory cytokines expression in DUSP4 KO BMDMs upon sendai virus and VSV infection. (A) Increased type I IFNs, IL-6 and TNFα expression in DUSP4 KO BMDMs upon sendai virus (MOI, 1) infection. (B) Increased type I IFNs, IL-6 and TNFα production in DUSP4 KO BMDMs upon VSV (MOI, 1) infection.

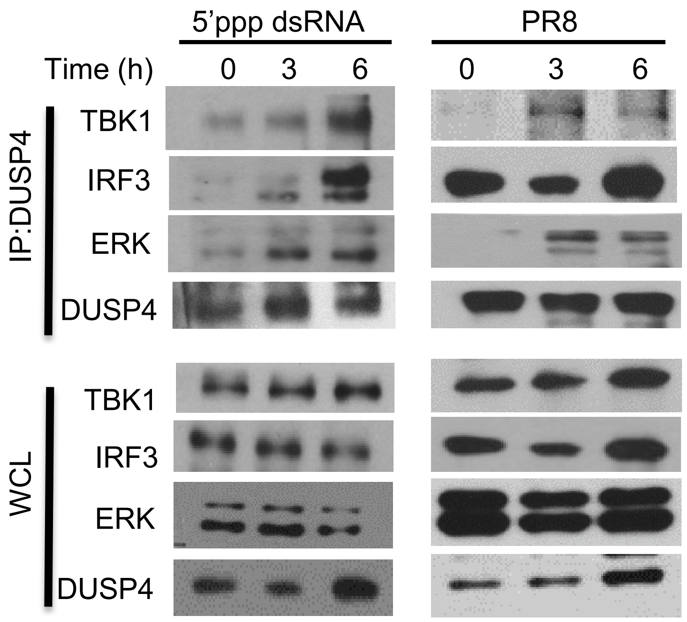

Figure S5. ***Endogenous DUSP4 interacts with endogenous TBK1, IRF3 and ERK.*** *Raw264.7 macrophages were infected with PR8 virus or stimulated with 5'ppp dsRNA. Cell lysates were precleared with A/G magnetic beads (Pierce) for 1 hr followed by incubation with anti-DUSP4 antibody (Abcam) overnight. After incubation, A/G beads were added to pull-down DUSP4 interacting proteins followed by western blot analysis to detect TBK-1,IRF3 and ERK1/2.*

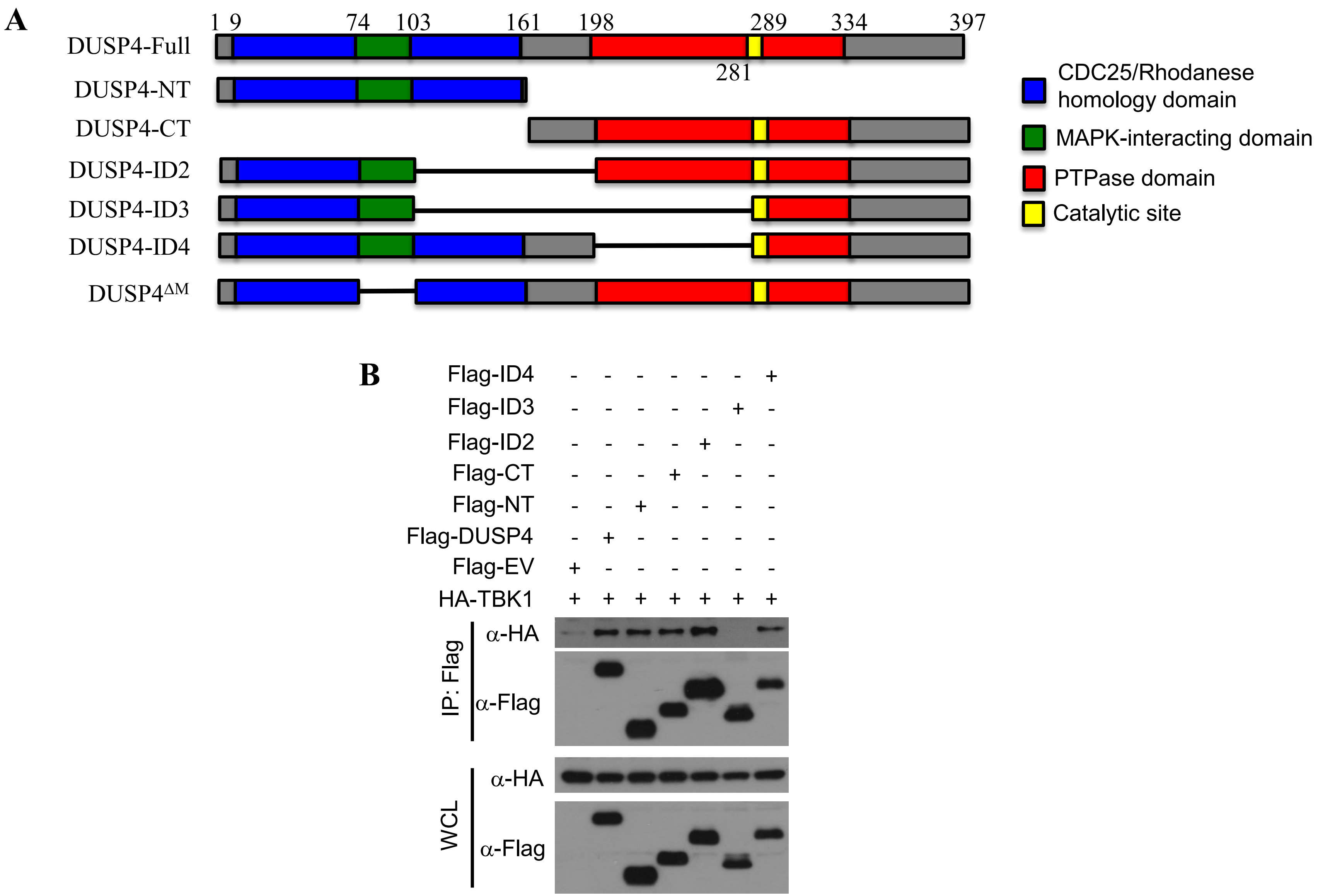

Figure S6 Internal domain 3 of DUSP4 is required for its interaction with TBK1. (A) Schematic representation of various DUSP4 constructs. (B) HEK293T cells were transfected with TBK1 and the indicated DUSP4 constructs to perform immunoprecipitation followed by immunoblot analysis to test their interaction.

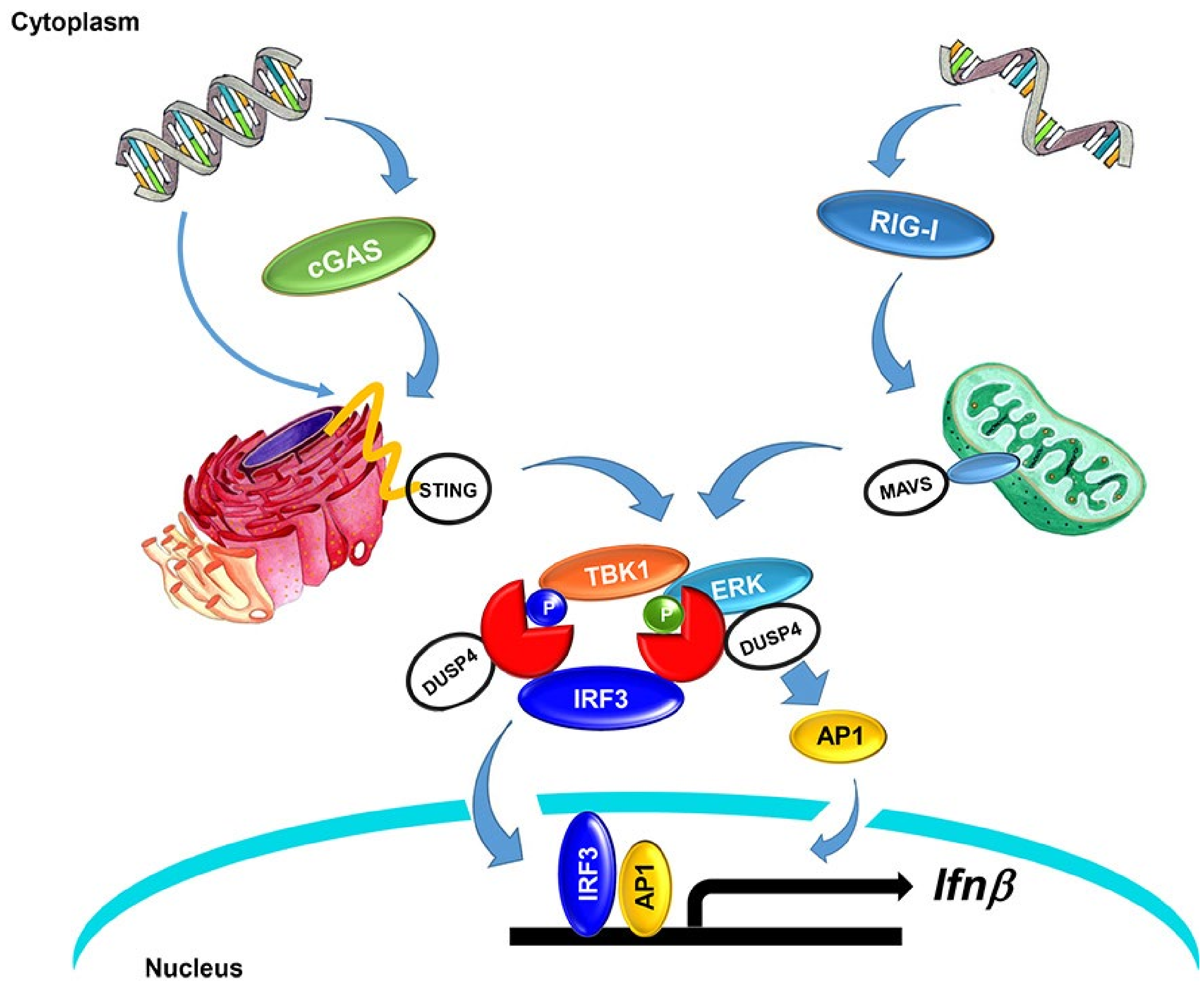

Figure S7. ***Regulatory function of DUSP4 in STING- and RIG-I-mediated type I interferon responses***. *In innate immune cells such as macrophages, cytosolic RNA and DNA from microbial pathogens are detected by RIG-I and cGAS respectively, which lead to the activation of TBK1-IRF3 and ERK for the transcription of IFNβ. Meanwhile, microbial pathogen infection increased the expression of DUSP4 which is recruited into a signalling complex including TBK1, ERK and IRF3. DUSP4 dephosphorylates TBK1 and ERK thereby downregulating the activation of IRF3 and AP1 to negatively regulate IFNβ expression.*
