# Supplementary figures and images for: DUSP4 modulates RIG-I- and STING-mediated IRF3-type I IFN response
Source: Cell Death Differ. 2024 Feb 21;31(3):280–91. doi: 10.1038/s41418-024-01269-7 (PMC10923883; doi:10.1038/s41418-024-01269-7)

1B

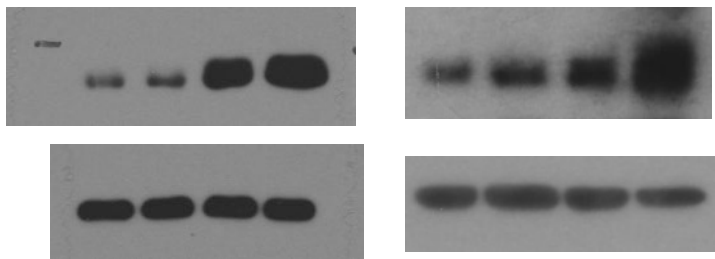

1G

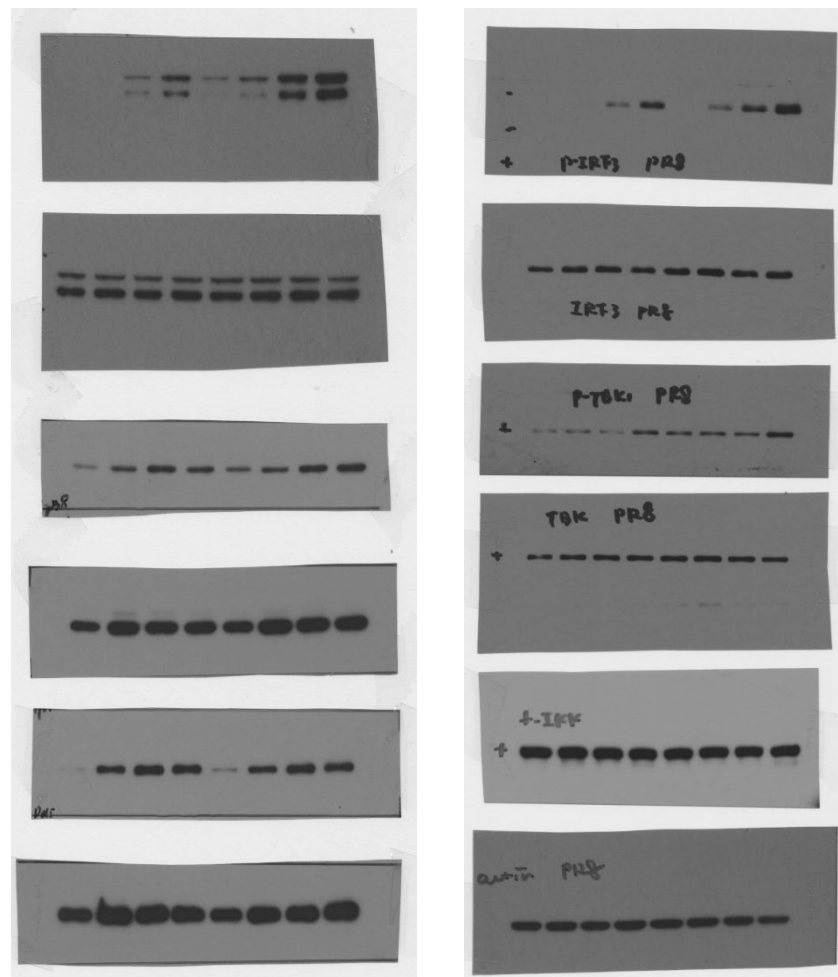

1D

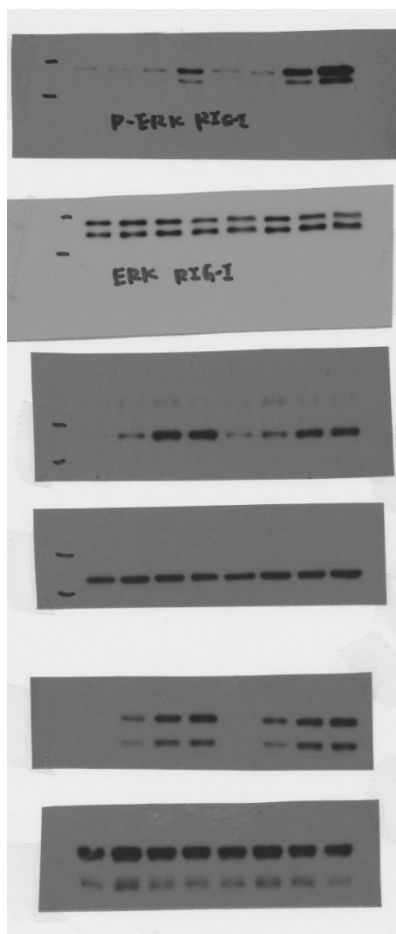

1E

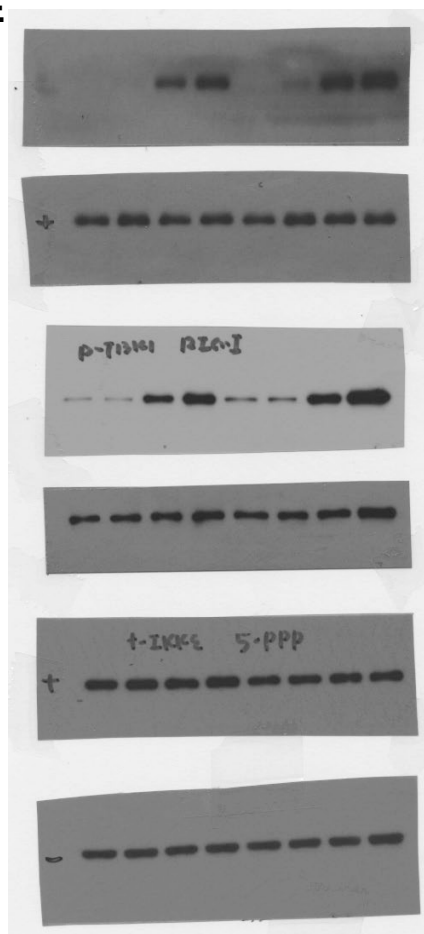

3B

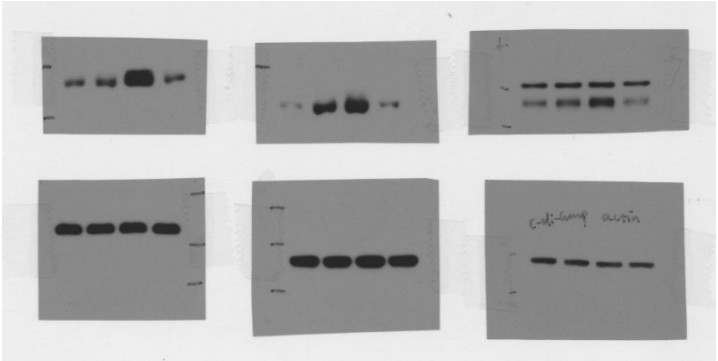

3D

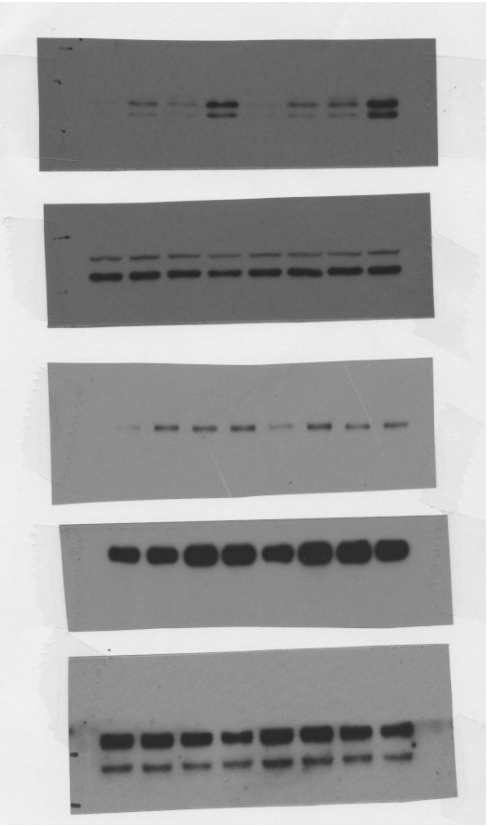

3F

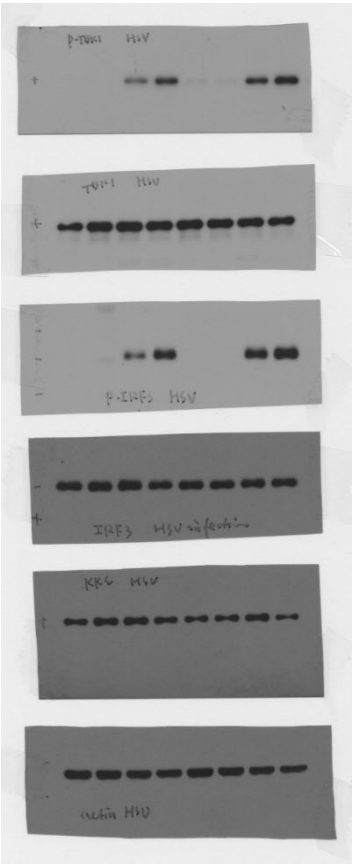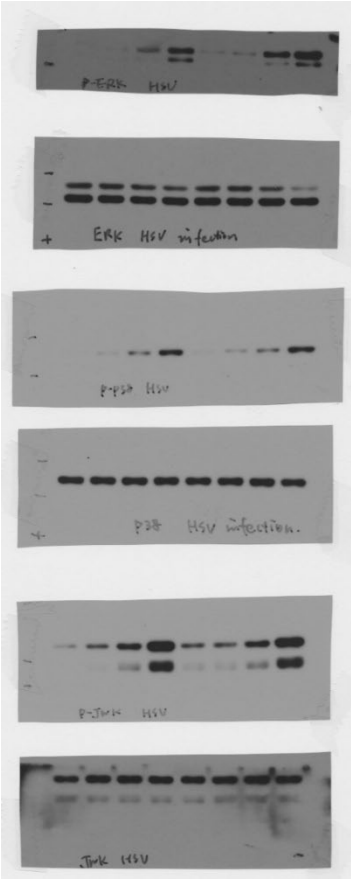

4B

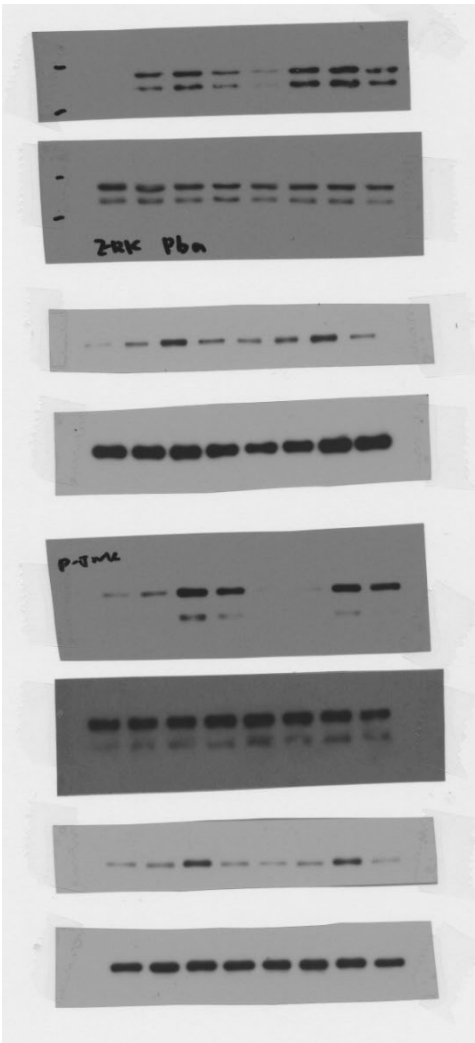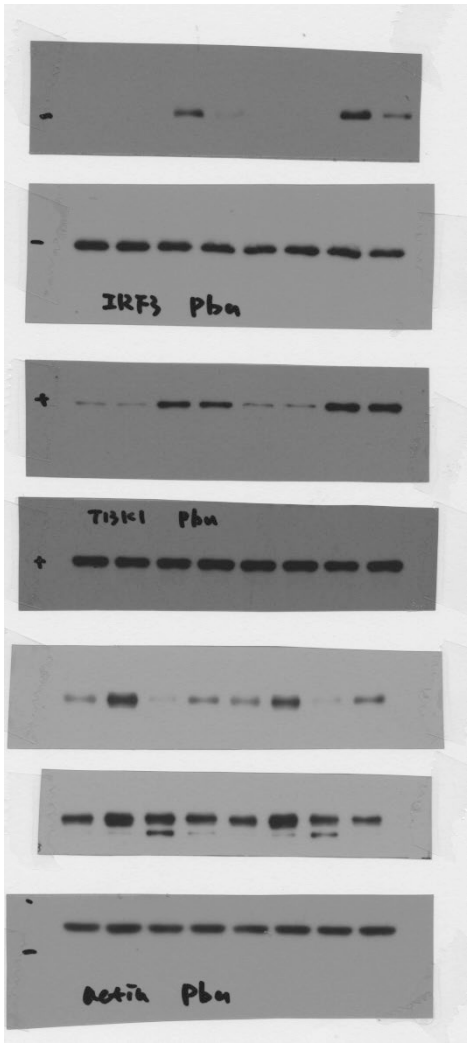

5A

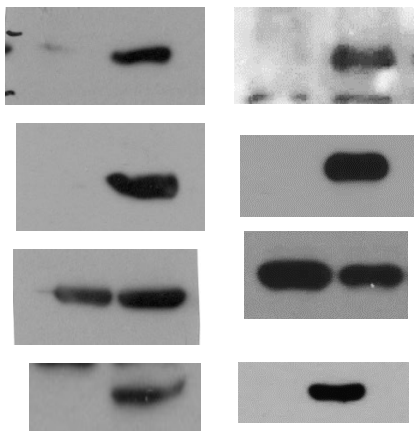

5B

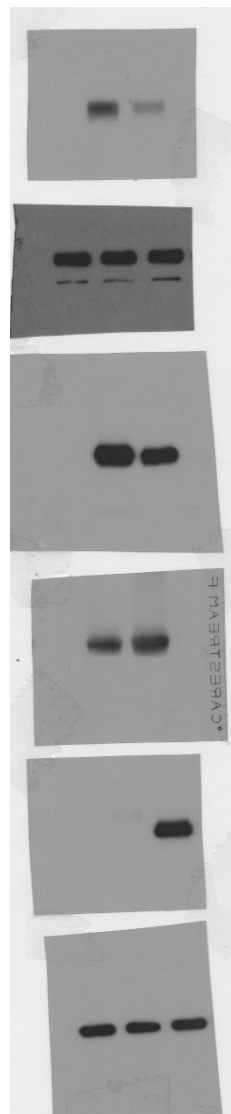

5C

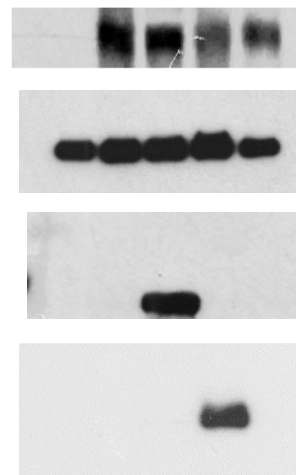

5D

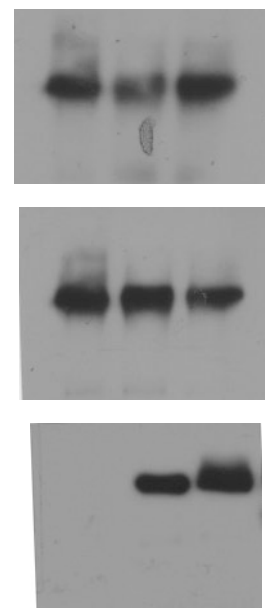

5E

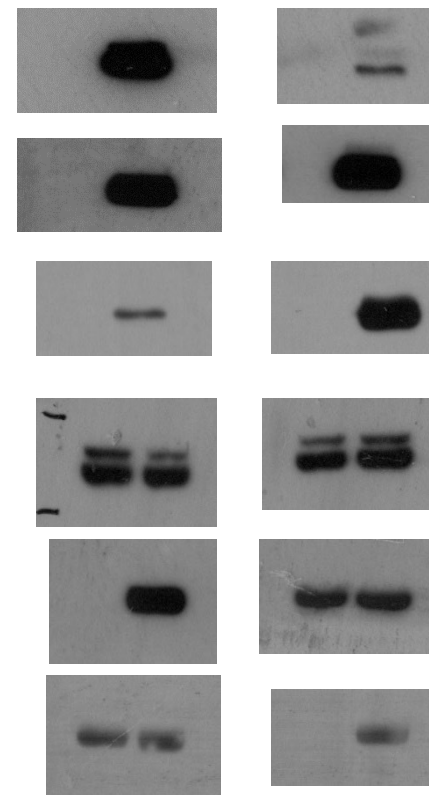

6A

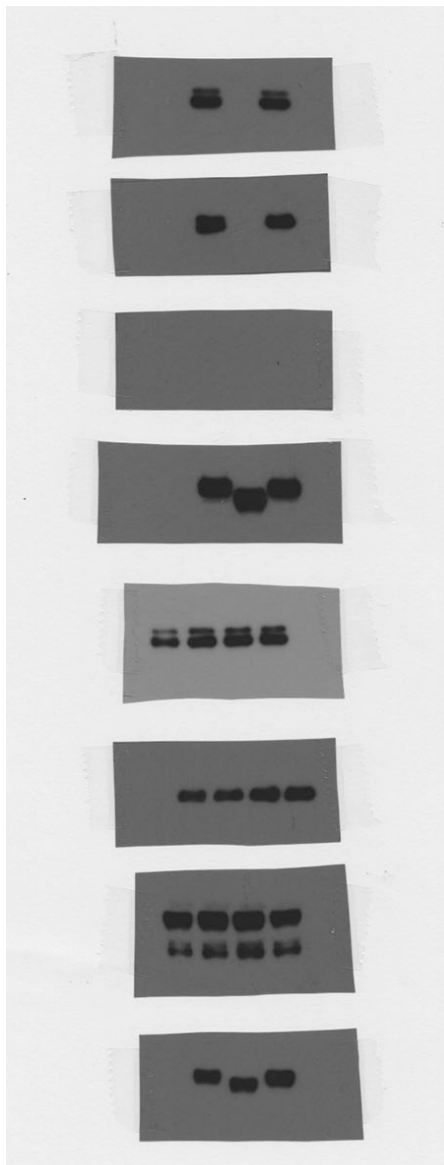

6B

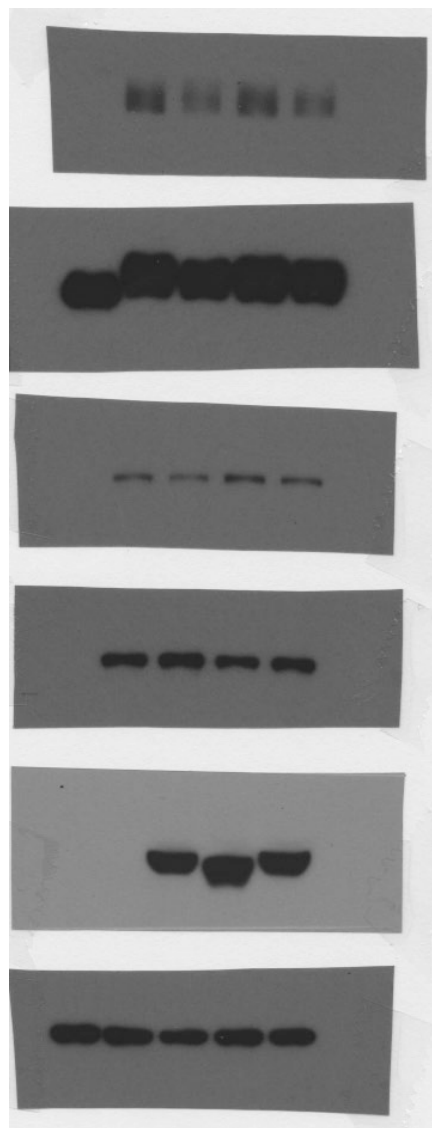

6C

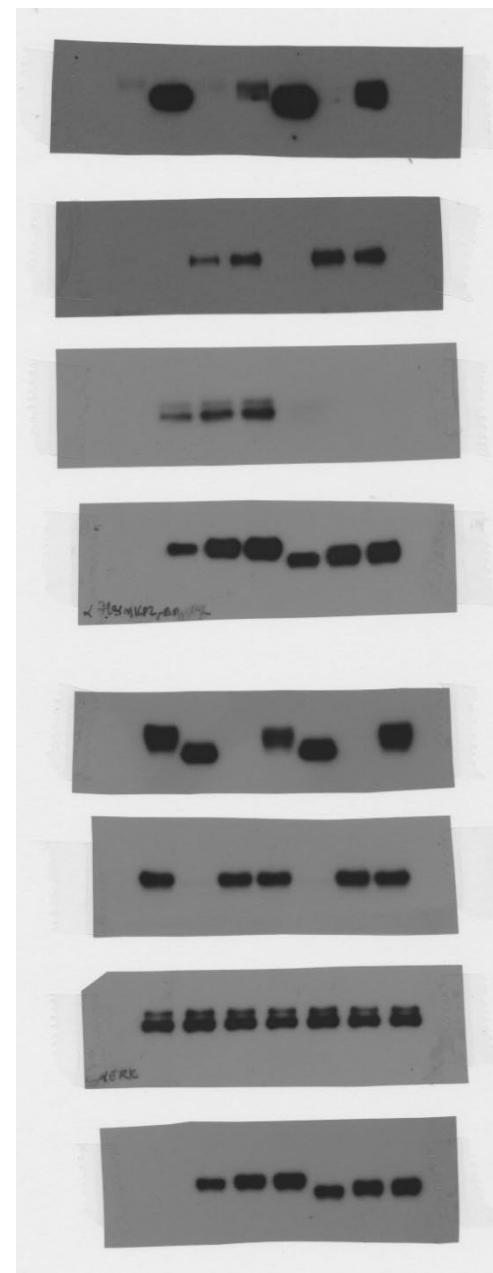



S2A

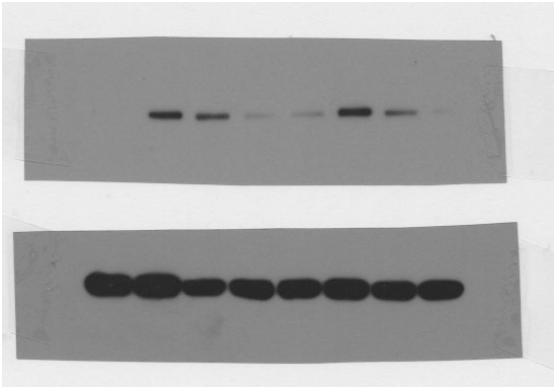

S2C

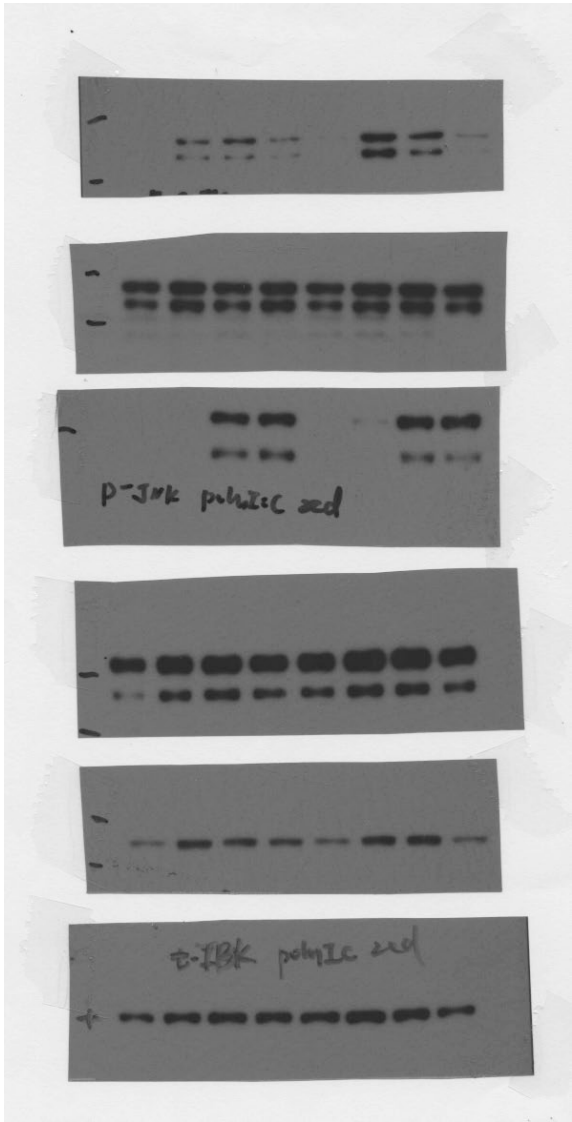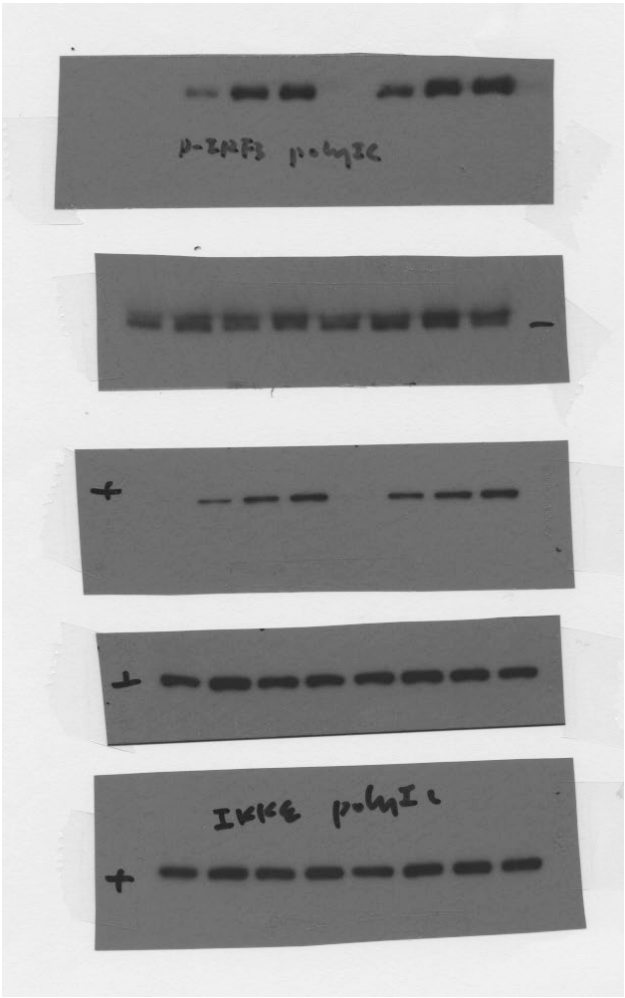

**S5**

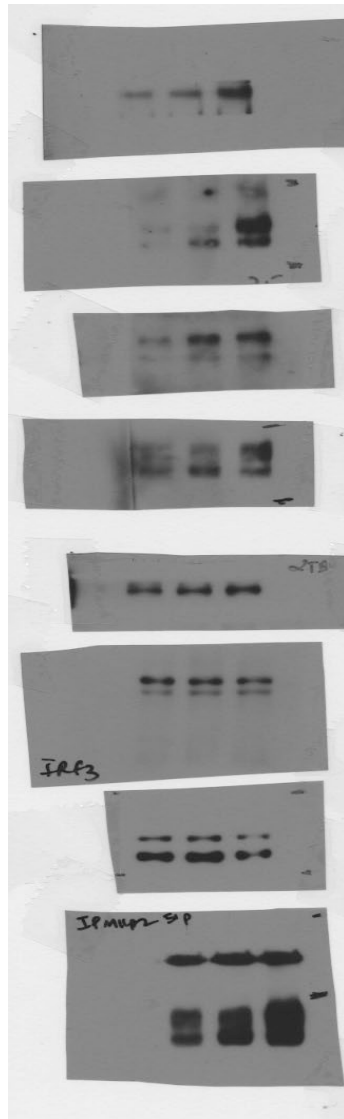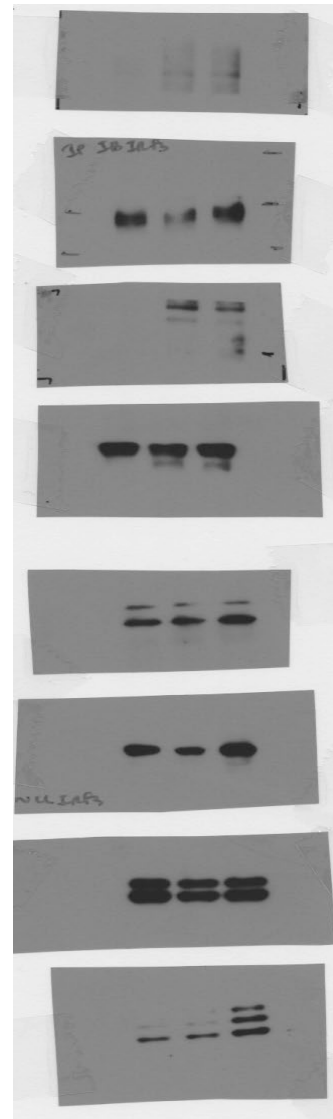

**S6B**

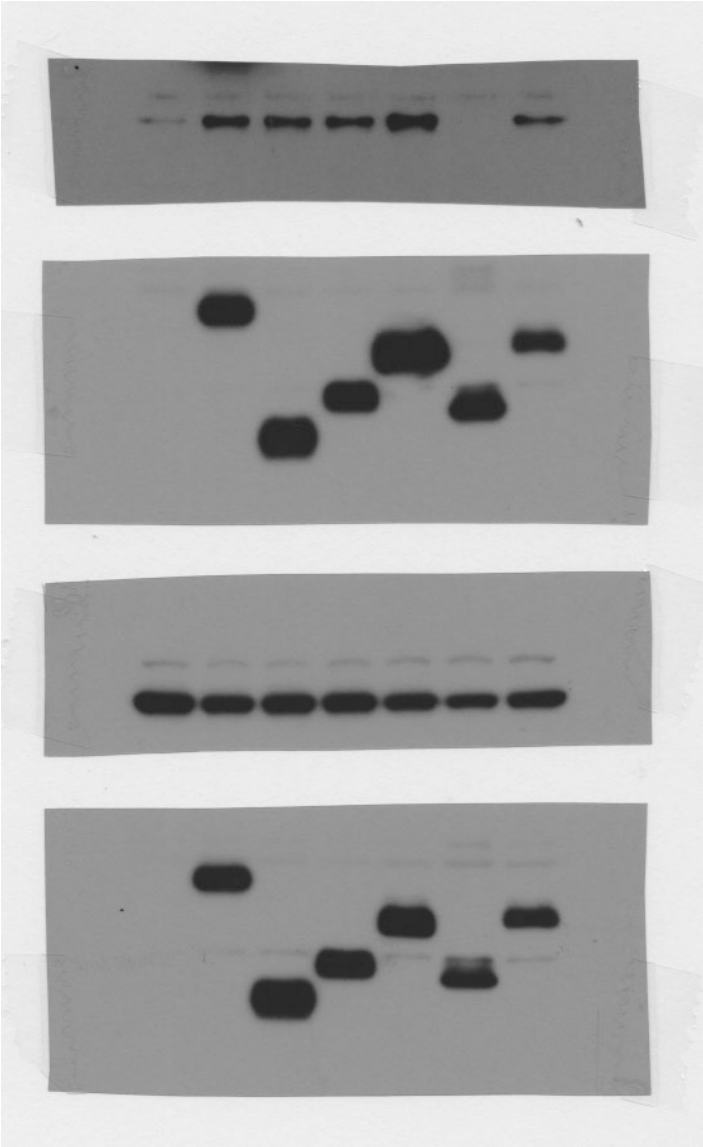

Supplement: Supplementary file 2 — Supplemental files [file 41418_2024_1269_MOESM2_ESM.pdf]
