# Supplementary material for: DUSP4 modulates RIG-I- and STING-mediated IRF3-type I IFN response
Source: Cell Death Differ. 2024 Feb 21;31(3):280–91. doi: 10.1038/s41418-024-01269-7 (PMC10923883; doi:10.1038/s41418-024-01269-7)
Supplement: Supplementary file 3 — Table S1 [file 41418_2024_1269_MOESM3_ESM.pdf]

Information on antibodies used in the study

Antibody	Cat # or Clone #	Dilution	Source
DUSP4	ab216576	1:1000	Abcam
ERK	4695	1:1000	Cell Signaling
Phospho-ERK	4377	1:1000	Cell Signaling
P38	9212	1:1000	Cell Signaling
Phospho-p38	9215	1:1000	Cell Signaling
JNK	9252	1:1000	Cell Signaling
Phospho-JNK	4671	1:1000	Cell Signaling
TBK1	3504	1:1000	Cell Signaling
Phospho-TBK1	5483	1:1000	Cell Signaling
IKKε	2690	1:1000	Cell Signaling
NFκB	8242	1:1000	Cell Signaling
Phospho-NFκB	3033	1:1000	Cell Signaling
IRF3	4302	1:1000	Cell Signaling
Phospho-IRF3	4947	1:1000	Cell Signaling
MKP5	3483	1:1000	Cell Signaling
HA	H6908	1:1000	Sigma-Aldrich
Flag	F7425	1:10000	Millipore
β-Actin	4970	1:1000	Cell Signaling
